# Cellular uptake of extracellular nucleosomes induces innate immune responses by binding and activating cGMP-AMP synthase (cGAS)

**DOI:** 10.1038/s41598-020-72393-w

**Published:** 2020-09-21

**Authors:** Huawei Wang, Chuanlong Zang, Mengtian Ren, Mengdi Shang, Zhenghua Wang, Xuemei Peng, Qiangzhe Zhang, Xin Wen, Zhen Xi, Chuanzheng Zhou

**Affiliations:** 1 State Key Laboratory of Elemento-Organic Chemistry and Department of Chemical Biology, College of Chemistry, Nankai University, Tianjin, 300071 China; 2 State Key Laboratory of Medicinal Chemical Biology and College of Pharmacy, Tianjin Key Laboratory of Molecular Drug Research, Nankai University, Tianjin, 300071 China

**Keywords:** Biochemistry, Chemical biology, Immunology

## Abstract

The nucleosome is the basic structural repeating unit of chromatin. DNA damage and cell apoptosis release nucleosomes into the blood circulatory system, and increased levels of circulating nucleosomes have been observed to be related to inflammation and autoimmune diseases. However, how circulating nucleosomes trigger immune responses has not been fully elucidated. cGAS (cGMP-AMP synthase) is a recently discovered pattern recognition receptor that senses cytoplasmic double-stranded DNA (dsDNA). In this study, we employed in vitro reconstituted nucleosomes to examine whether extracellular nucleosomes can gain access to the cytoplasm of mammalian cells to induce immune responses by activating cGAS. We showed that nucleosomes can be taken up by various mammalian cells. Additionally, we found that in vitro reconstituted mononucleosomes and oligonucleosomes can be recognized by cGAS. Compared to dsDNA, nucleosomes exhibit higher binding affinities to cGAS but considerably lower potency in cGAS activation. Incubation of monocytic cells with reconstituted nucleosomes leads to limited production of type I interferons and proinflammatory cytokines via a cGAS-dependent mechanism. This proof-of-concept study reveals the cGAS-dependent immunogenicity of nucleosomes and highlights the potential roles of circulating nucleosomes in autoimmune diseases, inflammation, and antitumour immunity.

## Introduction

The nucleosome is the basic structural repeating unit of chromatin, consisting of double-stranded DNA (dsDNA) wrapping around a histone octamer core^1^. Cell apoptosis induced by DNA damage or other lethal processes leads to chromosomal DNA fragmentation and the release of nucleosomes into the blood circulation system^2,3^. Increased circulating nucleosomes have been found to be related to inflammation and autoimmune diseases^4,5^. Thus, nucleosomes are considered to be a trigger of the immune response^6^. However, the mechanism by which nucleosomes induce immune and inflammatory responses has not been fully elucidated.

DNA fragments are pathogen-associated molecular patterns (PAMPs) and damage-associated molecular patterns (DAMPs) that can be recognized by pattern recognition receptors (PRRs) to initiate the immune response^7,8^. cGMP–AMP synthase (cGAS) is a PRR that senses cytoplasmic dsDNA derived from both pathogens and endogenous mitochondria/chromatin^9–11^. Binding of dsDNA to cGAS activates the latter to synthesize the second messenger 2′,5′-cyclic GMP–AMP (cGAMP)^10,12^, which is, in turn, sensed by stimulator of interferon genes (STING), thereby inducing the production of type I interferons (IFN-α, IFN-β) and/or pro-inflammatory cytokines such as IL-6, IL-8 and TNF^13,14^. Activation of the cGAS–STING pathway by cytoplasmic dsDNA leads to various biological consequences, such as innate immunity, inflammation-driven cell senescence, carcinogenesis and metastasis^15–19^.

Recently, several studies showed that cGAS binds directly to nucleosomes, activating itself to synthesize cGAMP^18,20,21^. The ability of nucleosomes to activate cGAS is considerably reduced relative to that of dsDNA. In the present study, we performed proof-of-concept studies to determine whether extracellular nucleosomes can gain access to the cytoplasm of mammalian cells to induce an immune response by activating the cGAS–STING pathway. Our results showed that nucleosomes, both reconstituted in vitro and isolated from cells, can be taken up by different types of mammalian cells in the absence of any delivery reagents. After cellular uptake, nucleosomes are bound by cGAS in the cytoplasm, triggering the production of type I interferons and proinflammatory cytokines through a cGAS-dependent mechanism.

## Results

### Preparation of nucleosomes

**Figure 1:**
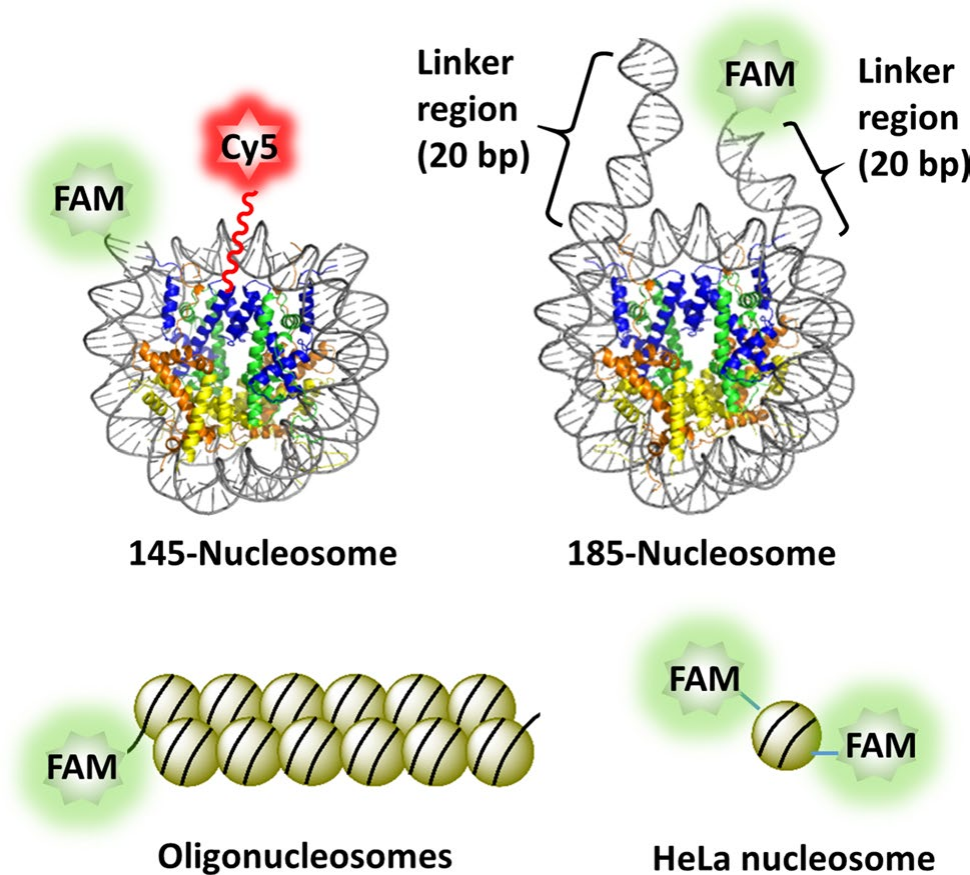
Structures of nucleosomes employed in the present study. For 145- and 185-nucleosome and oligonucleosomes, one FAM moiety was tagged on the 5′ end of one DNA strand. For the HeLa nucleosome, FAM was introduced to Lys residues of histones by chemical modification, and therefore, one HeLa nucleosome may contain multiple FAM moieties. For the dual labelled 145-nucleosome, one Cy5 fluorophore was tagged on Cys35 of each histone H3. Figures of 145-nucleosome and 185-nucleosome created using data from PDB: 3LZ0 and 1ZBB, respectively.

During apoptosis, caspase-activated DNase cleaves chromosomal DNA at the internucleosomal linkers to generate mononucleosomes and oligonucleosomes, with the former being predominant^22,23^. In this regard, we prepared reconstituted mononucleosomes and oligonucleosomes to examine their immunogenicity (Fig. 1). One such mononucleosome is the “601” nucleosome core particle (henceforth referred to as the 145-nucleosome), which was prepared by reconstitution of the 145 bp ‘‘Widom 601” DNA (145-dsDNA) with recombinant histone octamer. The 145-nucleosomes are well-positioned nucleosome core particles (NCPs) whose structure has been determined by X-ray diffraction^24,25^. We have demonstrated that in vitro reconstituted 145-nucleosomes are excellent models for mechanistic studies of DNA damage in nucleosomes^26–31^. Another type of mononucleosome employed in this study is the 185-nucleosome, which consists of 185 bp dsDNA (185-dsDNA) wrapped around the histone core. The 185-dsDNA was constructed by extending the 145 bp “Widom 601” DNA on both ends by 20 bp whose sequence was randomly designed. Hence, the 185-nucleosome, having two linker dsDNA regions that stretch out and are unshielded by the histone core (Fig. 1)^32^, was used to examine the effect of the linker dsDNA on cGAS activation. In addition, we prepared an oligonucleosome substrate by reconstituting one 2,450 bp DNA (2,450-dsDNA) molecule with histone octamers. The 2,450-dsDNA was PCR amplified from a plasmid whose sequence was randomly designed, and the obtained oligonucleosomes were supposed to be mixtures of 12–14-mer oligonucleosome arrays (Fig. 1)^33^.

The 145-nucleosome, 185-nucleosome and oligonucleosomes prepared by in vitro reconstitution contain no modifications on DNA and histones. In this regard, the nucleosomes are slightly different from circulating mononucleosomes inside the body. To examine the effect of “real” circulating nucleosomes, we also prepared HeLa nucleosomes by isolating the chromatin of HeLa cells followed by digestion with micrococcal nuclease. After purification by gel filtration, the obtained HeLa nucleosomes were primarily composed of mononucleosomes that resemble the 145-nucleosome (Supplementary Fig. S1).

### Cellular entry of nucleosomes

cGAS is a cytoplasmic receptor. To trigger the immune response by activating cGAS, extracellular nucleosomes must penetrate the cellular membrane and gain access to the cytoplasm. Therefore, we examined the cellular uptake efficiency of nucleosomes using fluorescence-labelled nucleosomes. To this end, 145-nucleosomes, 185-nucleosomes and oligonucleosomes were reconstituted using 5′-fluorescein (FAM)-labelled 145-dsDNA, 185-dsDNA and 2,450-dsDNA, respectively, and isolated HeLa nucleosomes were chemically labelled through the reaction of 5(6)-carboxyfluorescein *N*-hydroxysuccinimide ester (FAM-NHS) with Lys residues of histones. Therefore, each reconstituted nucleosome had one FAM group attached on the 5′ end of one DNA strand, while the nucleosome isolated from HeLa cells could have more than one FAM label (Fig. 1).

**Figure 2:**
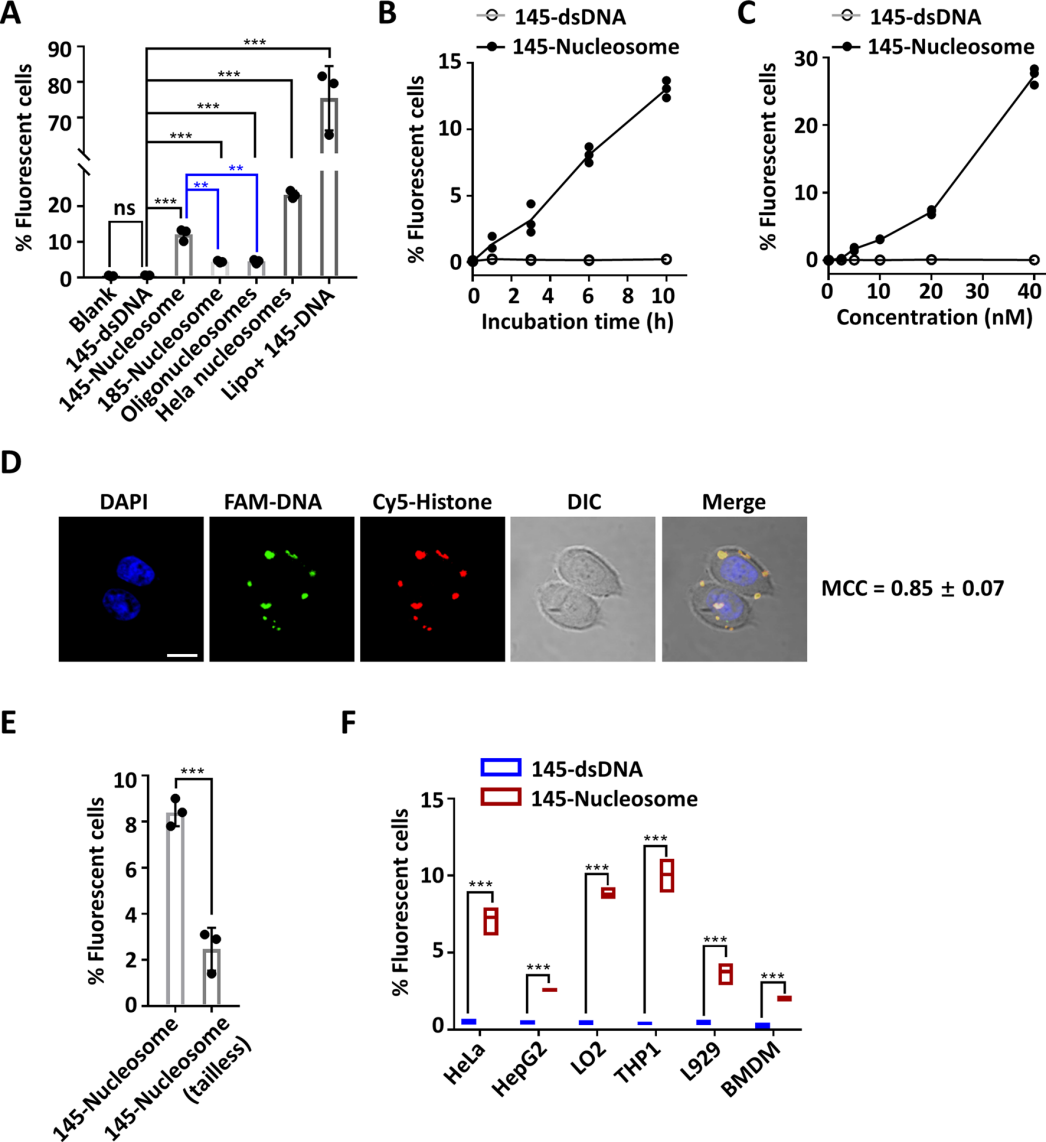
Cellular entry of nucleosomes. (**A**) Flow cytometry analysis of the uptake of free DNA and nucleosomes by HeLa cells. HeLa cells were incubated with 145-dsDNA or nucleosomes (20 nM) for 10 h. (**B**) Flow cytometry analysis of the time-dependent uptake of 145-nucleosomes (20 nM) by HeLa cells. (**C**) Flow cytometry analysis of the concentration-dependent uptake of 145-nucleosomes (after 6 h incubation) by HeLa cells. (**D**) Representative images showing the cellular location of 145-nucleosomes in HeLa cells after incubation for 12 h. FAM was tagged on the 5′ end of one DNA strand, and Cy5 was conjugated to Histone H3. Scale bar, 20 μm. MCC (Manders' colocalization coefficient) is the ratio of the FAM signal that overlaps with the Cy5 signal to the total signal of FAM. (**E**) Flow cytometry analysis of the uptake of tailless 145-nucleosomes (20 nM) by HeLa cells (after 6 h incubation). Tailless 145-nucleosomes contain tailless histone mutants, H4-del 1–20, H3-del 1–37, H2A-del 1–15 and H2B-del 1–31, instead of wild type histones. (**F**) Flow cytometry analysis of cellular uptake of 145-nucleosomes (20 nM) to different cell lines after 6 h incubation. For all graphs, error bars indicate the mean ± standard deviation of three independent experiments. Statistical significance was determined based on Student’s *t*-test (ns, p > 0.05; *0.05 > p > 0.01; **0.01 > p > 0.001; ***0.001 > p).

Cellular uptake of nucleosomes was first examined using HeLa cells. HeLa cells were cultured in Opti-MEM medium containing FAM-labelled 145-dsDNA or nucleosomes (20 nM) for 10 h. Flow cytometry analyses of the cultured cells revealed that 1%, 12%, 5%, 5% and 23% of cells were fluorescent for culture with 145-dsDNA, 145-nucleosomes, 185-nucleosomes, oligonucleosomes and HeLa nucleosomes, respectively (Fig. 2A). These results confirmed that all of the different types of nucleosomes can be taken up by HeLa cells, although the cellular uptake of nucleosomes was less efficient than the delivery of 145-dsDNA using Lipofectamine 2000 (76%). Comparatively, 185-nucleosomes and oligonucleosomes with free linker DNA on both ends showed lower cellular uptake than 145-nucleosomes. The ratio of fluorescent cells upon treatment with HeLa nucleosomes (23%) was approximately twofold that with 145-nucleosomes (12%). However, given that each HeLa nucleosome may contain more than one FAM label, this observation may not suggest that HeLa nucleosomes are more permeable than the in vitro reconstituted, unmodified nucleosomes.

The efficiency of cellular uptake of 145-nucleosomes by HeLa cells was dependent on both the incubation time and nucleosome concentrations. Increasing the incubation time led to increased entry efficiency (Fig. 2B), and the same finding was observed for increasing the nucleosome concentrations (Fig. 2C). Up to 26% of HeLa cells contained 145-nucleosomes after incubation with 40 nM of the 145-nucleosomes for 6 h.

Next, confocal fluorescence microscopy was employed to examine the cellular distribution of entry nucleosomes. After incubation with different types of FAM-labelled nucleosomes for 6 h, the fluorescence of FAM was mainly observed in the cytoplasm of HeLa cells (Fig. S2). Immunofluorescence staining of EEA1, an early endosome marker, revealed that nucleosomes were not localized in endosomes (Manders' colocalization coefficient, MCC ≈ 0.1, Fig. S3). However, this finding does not rule out the possibility that nucleosomes are located in other intracellular vesicles. Furthermore, dual labelled 145-nucleosomes were prepared to verify whether the nucleosomes retained integrity after cellular entry. To this end, a histone H3 mutant H3-C110A-V35C, which contains only one Cys at position 35, was tagged with one Cy5 fluorophore via Michael addition of Cys35 to Cy5-maleimide. Then, the obtained Cy5-H3 was employed with three other histones to prepare the Cy5-labelled histone octamer. Reconstitution of FAM-labelled 145-DNA with the Cy5-labelled histone octamer generated the desired nucleosomes with FAM and Cy5 dual fluorescence labelling^34^. After incubating HeLa cells with the dual labelled nucleosomes for 12 h, confocal fluorescence microscopy analysis showed that FAM and Cy5 colocalized notably well in the cytoplasm, showing a MCC of 0.85 ± 0.07 (Fig. 2D). Taken together, these results indicated that nucleosomes are taken up by cells as intact complexes and retain integrity after cellular entry.

Free dsDNA molecules generally show no cellular uptake because the negatively charged phosphate backbone hampers their penetration of the negatively charged cellular membrane. In contrast, histones are cellular permeable because they are Lys- and Arg-rich, especially in their N-terminal tails, and thus are positively charged under physiological conditions^35,36^. In nucleosomes, the neutralization of negative charges of DNA by positively charged histone tails is thus supposed to account for the increased cellular permeability of nucleosomes compared to dsDNA. To prove this hypothesis, we prepared tailless 145-nucleosomes in which four wild type histones are replaced by tailless histone mutants, H4-del 1–20, H3-del 1–37, H2A-del 1–15 and H2B-del 1–31. The cellular uptake efficiency of the tailless 145-nucleosomes was only 1/4 of that of wild type 145-nucleosomes (Fig. 2E), highlighting the key role of positively charged histone tails in the efficient cellular uptake of nucleosomes.

Except for HeLa cells, a broad range of mammalian cell lines, including the human cancer cell line HepG2, normal cell line LO2, monocytic cell line THP1, murine cell line L929 and murine bone marrow-derived macrophage (BMDM) cells, could take up nucleosomes. The uptake efficiency varied to some extent in different cell lines (Fig. 2F).

### Nucleosomes bind cGAS to trigger the synthesis of cGAMP in vitro

Depending on the length of dsDNA, cGAS binds dsDNA to form different types of cGAS-DNA complexes: the minimal cGAS_1_–DNA_1_ complex for short dsDNA and oligomer cGAS_2n_–DNA_2_ (n ≥ 1) complex for longer dsDNA^37–41^. dsDNA binding induces the structural switch of cGAS, thereby activating the latter to synthesize the second messenger cGAMP. Recent studies have shown that cGAS also binds to mononucleosome^18,20,21^. To examine whether different types of nucleosomes can be recognized and bound by cGAS, 5′-FAM-labelled 145-nucleosomes, 185-nucleosomes, oligonucleosomes and 145-tailless nucleosomes were incubated with different concentrations of human cGAS (Supplementary Fig. S4). Electrophoretic mobility shift assays (EMSAs) of the reactions showed that all of the nucleosomes were bound by cGAS to form higher-order complexes that stayed at the top of the gel (Supplementary Figs. S5–S8). Plotting the percentages of bound nucleosomes against the concentrations of cGAS yielded the dissociation constants (Table 1)^42^. Consistent with previous reports^38,40,43^, cGAS showed a higher affinity for the longer 2,450-dsDNA than for the short 145-dsDNA. However, similar *K*_d_ values were observed for the binding of cGAS to oligonucleosomes and 145-mononucleosomes. Thus, unlike binding dsDNA, binding nucleosomes by cGAS is not dependent on the DNA length.

**Table 1:** Dissociation constants (K_*d*_) of interactions between human cGAS and different substrates.

Substrates	*K*_d_ (nM)
145-dsDNA	350 ± 37
145-Nucleosome	67 ± 5
185-dsDNA	394 ± 38
185-Nucleosome	172 ± 21
2,450-dsDNA	68 ± 4
Oligonucleosomes	66 ± 6
145-Tailless nucleosome	79 ± 7

Comparatively, cGAS showed higher binding affinity to 145-nucleosome and 185-nucleosome than 145-dsDNA and 185-dsDNA, respectively, but it exhibited unnoticeable differences in binding 2,450-dsDNA and oligonucleosomes. Therefore, nucleosomes are possibly better, and at least not worse, substrates for cGAS binding relative to their dsDNA counterparts. Interestingly, cGAS showed highly similar K_*d*_ values to 145-nucleosome and 145-tailless nucleosome, suggesting that histone tails are not fundamental for cGAS binding.

Given that nucleosomes are formed by the non-covalent interactions between dsDNA and the histone core, cGAS may strip dsDNA from nucleosomes to form cGAS–dsDNA complexes lacking histones. To rule out this possibility, 5′-FAM-DNA and Cy5-H3 dual labelled 145-nucleosomes were employed for incubation with cGAS followed by EMSA analysis. FAM and Cy5 fluorescence imaging of the gel revealed that nucleosomes retained integrity in the formed complexes (Fig. S9). Therefore, the nucleosome, as a whole, can be efficiently recognized and bound by cGAS.

**Figure 3:**
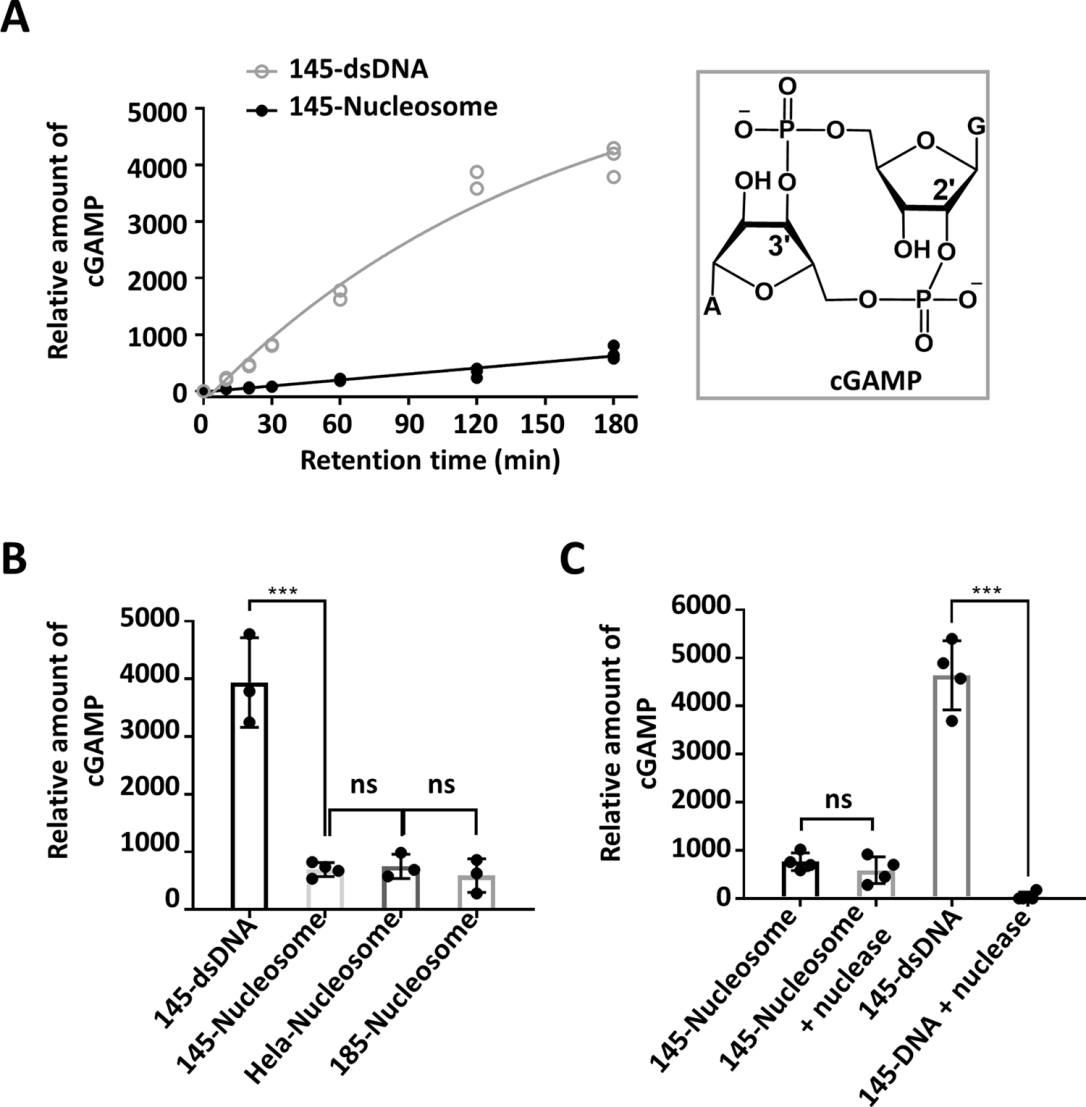
In vitro analyses of cGAMP generation upon incubation of cGAS with dsDNA or nucleosomes. (**A**) Kinetics of cGAMP production induced by 145-nucleosomes and 145-dsDNA. (**B**,**C**) Comparison of cGAMP production after incubation of cGAS with different dsDNA and nucleosomes for 3 h. Reaction conditions: dsDNA or nucleosomes (80 ng DNA/μl) were mixed with human cGAS (2 μM) in a Tris–HCl buffer (20 mM, pH 7.6, 60 mM NaCl, 2.5 mM ATP, 2.5 mM GTP, 5 mM MgCl_2_). After incubation at 37 °C, aliquots were taken and analysed by UPLC-MS. For all graphs, error bars indicate the mean ± standard deviation of three independent experiments. Statistical significance was determined based on Student’s *t*-test (ns, p > 0.05; *0.05 > p > 0.01; **0.01 > p > 0.001; ***0.001 > p).

Next, activation of cGAS by nucleosomes to synthesize cGAMP was examined in vitro based on UPLC-MS/MS analyses. Incubation of both 145-dsDNA and 145-nucleosome with cGAS in the presence of ATP and GTP produced cGAMP (Fig. S10). The amount of cGAMP accumulated with time (up to 3 h), and the initial velocity of cGMAP generation induced by 145-nucleosome was only 1/10 of that induced by 145-dsDNA (Fig. 3A). Under the same conditions, different types of nucleosomes exhibited the ability to activate cGAS. After incubation with cGAS in the presence of ATP and GTP for 3 h, 185-nucleosomes and HeLa nucleosomes produced similar amounts of cGAMP as 145-nucleosomes, which was approximately 15% of that induced by dsDNA (Fig. 3B). Taken together, these results indicate that although nucleosomes showed even higher binding affinities to cGAS than dsDNA, the former are considerably less potent in activating cGAS than the latter.

To preclude the possibility that nucleosome-triggered cGAMP production arose from the trace amount of free dsDNA remaining in the nucleosome samples, 145-nucleosomes were treated with micrococcal nuclease to digest free dsDNA and the linker DNA of nucleosomes before cGAS activation. However, cGAMP production was not affected (Fig. 3C). In contrast, treatment of 145-dsDNA with micrococcal nuclease completely abolished cGAMP production. Taken together, these in vitro results suggested that nucleosomes can be recognized and bound by cGAS and trigger cGAS to synthesize the second messenger cGAMP. Compared to 185-nucleosomes, 145-nucleosomes showed an even higher binding affinity to cGAS and exhibited similar potency in cGAS activation; thus, cGAS must bind not on the linker region but on the core region of nucleosomal DNA.

### Cellular entry of nucleosomes triggers the expression of INFβ and IL-6 via cGAS activation

**Figure 4:**
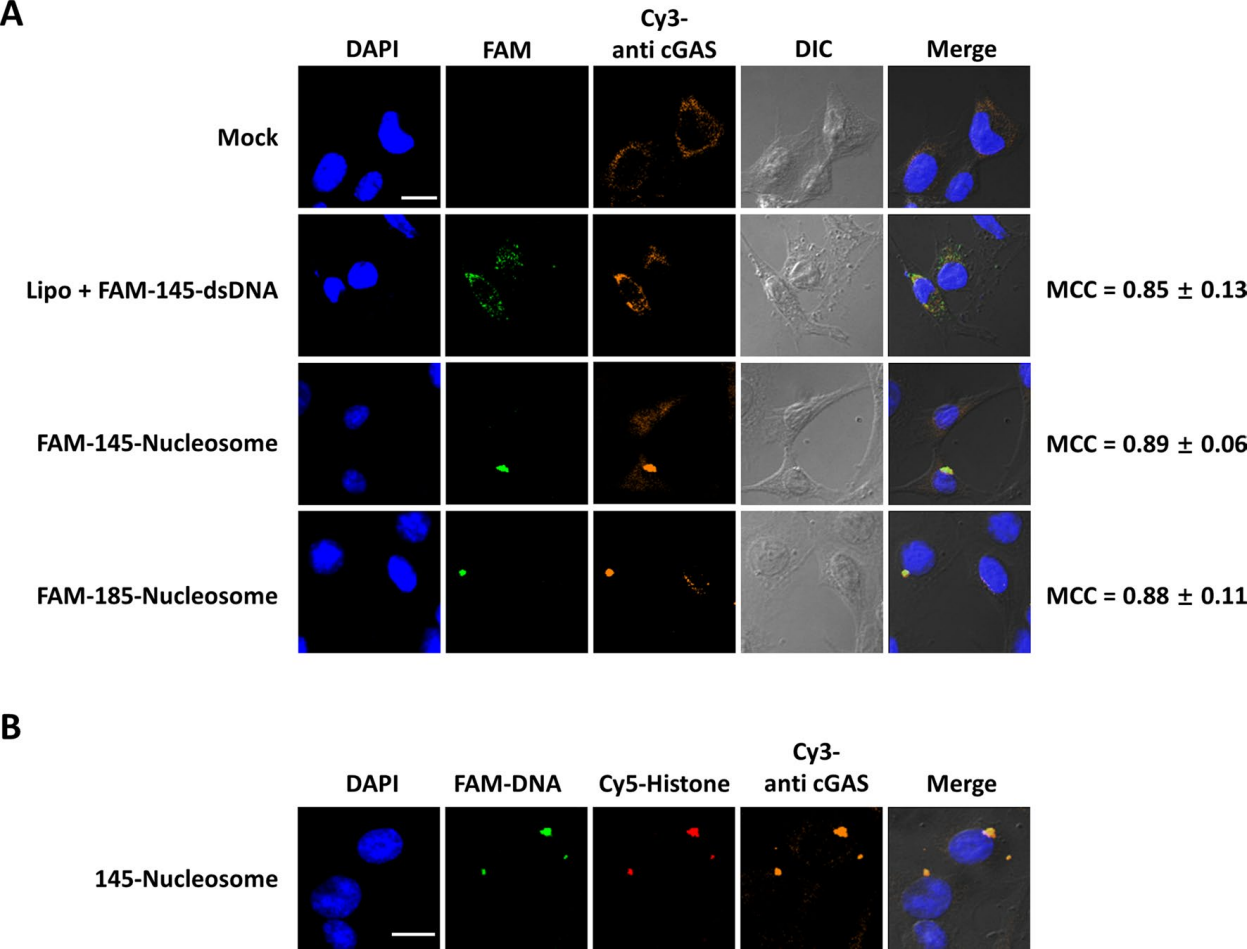
Confocal fluorescence microscopy analyses of the cellular location of nucleosomes and cGAS in L929 cells. (**A**) Representative images showing the cellular location of dsDNA, nucleosomes and cGAS after incubation for 12 h. MCC (Manders' colocalization coefficient) is the ratio of FAM signal that overlaps with Cy3 signal to the total signal of FAM. (**B**) Representative images showing the cellular location of FAM and Cy5 dual labelled 145-nucleosomes and cGAS after incubation for 12 h. FAM was tagged on the 5′ end of one DNA strand and Cy5 was conjugated in histone H3. Cy3 labelled secondary antibody was used for the immunofluorescence of cGAS. Scale bar, 20 μm.

Two cell lines stably expressing cGAS and STING—the murine fibrosarcoma cell line L929 and the human monocytic cell line THP1—were employed to examine whether entry of nucleosomes can activate cGAS to trigger innate immune responses in living cells. To this end, L929 cells were incubated with 5′-FAM-labelled 145-nucleosomes and 185-nucleosomes for 12 h. After cell fixation, confocal immunofluorescence microscopy analysis using anti-cGAS primary antibody and Cy3-labelled secondary antibody revealed that nucleosomes were colocalized with cGAS (MCC = 0.89 ± 0.06) in punctate forms in the cytoplasm (Fig. 4A). Notably, in cGAS knockout L929 (L929-*Cgas*^−/−^) cells, nucleosomes also formed large puncta (Supplementary Fig. S11). Thus, nucleosome aggregation in the cytoplasm is likely not dependent on the cGAS binding. In addition, the well colocalized of FAM and Cy5 dual labelled 145-nucleosomes with cGAS in L929 cells confirmed that cGAS directly binds to intact nucleosomes in the cytoplasm (Fig. 4B).

**Figure 5:**
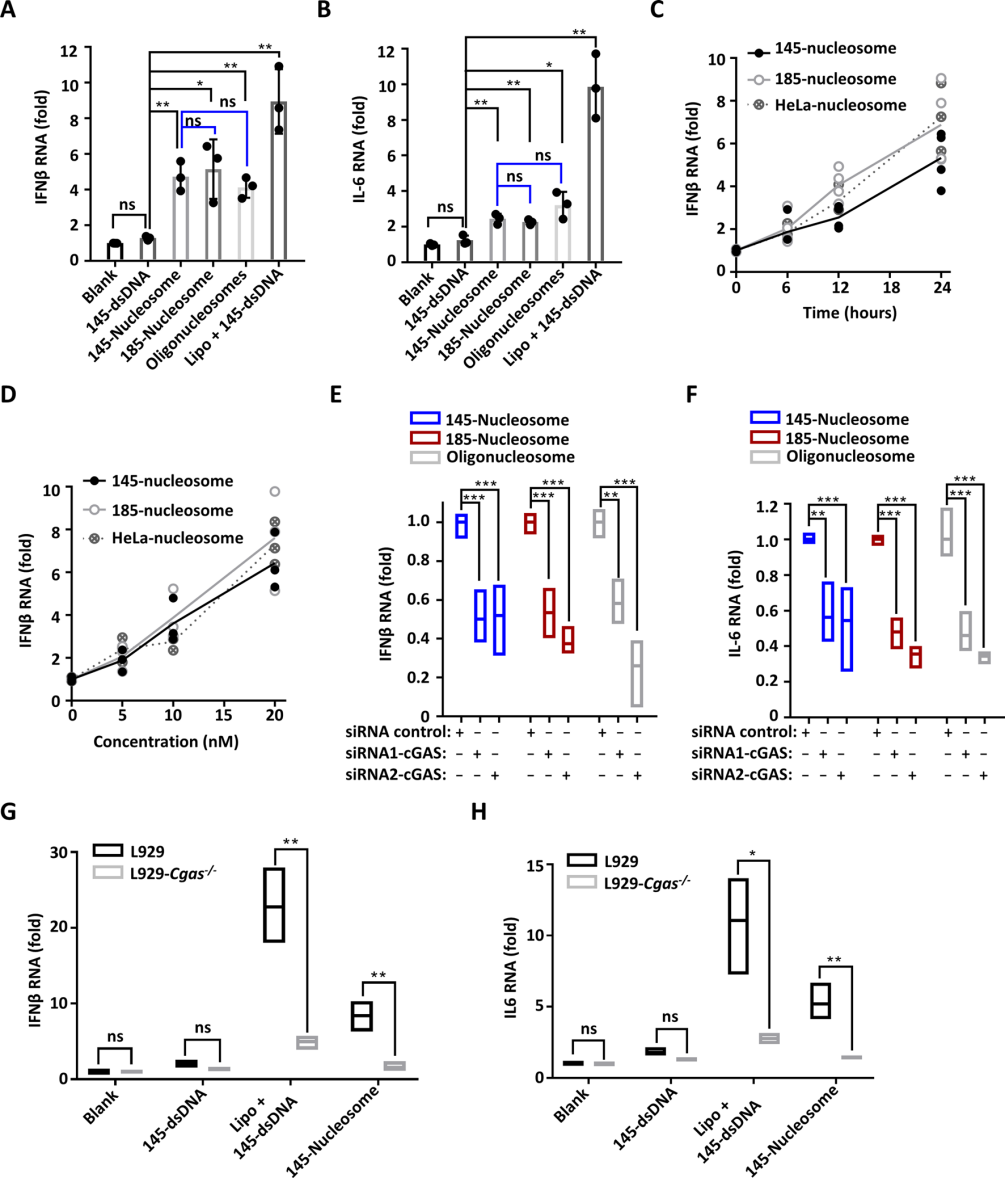
Cellular uptake of nucleosomes stimulates the secretion of INFβ and IL-6 through cGAS activation. (**A**,**B**) qRT-PCR analyses of IFNβ and IL-6 expression in THP1 cells upon incubation with different types of nucleosomes (20 nM) for 24 h. (**C**) qRT-PCR analyses of the time-dependent IFNβ expression in THP1 cells upon incubation with different types of nucleosomes (20 nM). (**D**) qRT-PCR analyses of the concentration-dependent IFNβ expression in THP1 cells upon incubation with different types of nucleosomes for 24 h. (**E**,**F**) qRT-PCR analyses of nucleosome-induced IFNβ and IL-6 expression in THP1 cells after cGAS knockdown by siRNAs. After treatment with siRNAs for 24 h, THP1 cells were incubated with 20 nM nucleosomes for 24 h, and secreted IFNβ and IL-6 in the cell culture media were quantified by ELISA. (**G**,**H**) qRT-PCR analyses of IFNβ and IL-6 expression in WT and cGAS knockout L929 cells upon incubation with different substrates (20 nM) for 24 h. For all graphs, error bars indicate the mean ± standard deviation of at least triplicate measurements. Statistical significance was determined based on Student’s *t*-test (ns, p > 0.05; *0.05 > p > 0.01; **0.01 > p > 0.001; ***0.001 > p).

Next, the induction of type I IFN and pro-inflammatory cytokines (such as IL-6) by the internalized nucleosomes was assessed in the human monocytic cell line THP1. Quantitative reverse transcription polymerase chain reaction (qRT-PCR) analyses showed that delivery of 145-dsDNA using Lipofectamine 2000 into THP1 cells led to a ninefold increase in both IFNβ and IL-6 RNA levels (Fig. 5A,B). However, incubation with 145-dsDNA omitting the delivery reagent produced no clear effect, confirming that extracellular free dsDNA cannot gain access to the cytoplasm to induce immune responses. Incubating THP1 cells with reconstituted nucleosomes resulted in approximately four to fivefold increases in IFNβ expression and two to threefold increases in IL-6 expression. Quantifying the secreted IFNβ and IL-6 based on enzyme-linked immunosorbent assay (ELISA) revealed a similar trend (Supplementary Fig. S12A,B). Thus, extracellular nucleosomes can trigger innate immune responses, and they are clearly less potent than dsDNA that delivered by lipofectamine.

qRT-PCR analyses showed that the levels of IFNβ expression in THP1 cells were almost linearly correlated with the concentrations of nucleosomes and incubation time (Fig. 5C,D). Obvious IFNβ induction was observed when THP1 cells were incubated with nucleosomes even at concentrations as low as 5 nM, which highlights the potency of extracellular nucleosomes in inducing the innate immune response. In addition, not only reconstituted 145-nucleosomes and 185-nucleosomes but also isolated HeLa nucleosomes exhibited highly similar efficiency in promoting IFNβ expression under the same conditions.

To examine whether nucleosome-triggered IFNβ and IL-6 expression in THP1 cells occurs via cGAS activation, two different pairs of small interfering RNAs (siRNAs) were introduced into THP1 cells to knock down cGAS, followed by incubation with different types of nucleosomes. Quantification of IFNβ and IL-6 expression based on both qRT-PCR and ELISA revealed that both siRNAs led to more than 50% inhibition of IFNβ and IL-6 expression (Fig. 5E,F, Supplementary Fig. S12C–E). Furthermore, 145-dsDNA and 145-nucleosome-induced IFNβ and IL-6 expression was examined in both WT L929 and L929-*Cgas*^−/−^ cells based on qRT-PCR. Under the same conditions, 4 to fivefold decreases in both IFNβ and IL-6 RNA levels were observed in the L929-*Cgas*^−/−^ cells relative to those in WT L929 cells (Fig. 5G,H, Supplementary Fig. S12F). In particular, after incubation of L929-*Cgas*^−/−^ cells with 145-nucleosomes, IFNβ and IL-6 expression was marginal, close to the basic level in WT L929 cells. This finding confirmed that cGAS activation is the main underlying mechanism governing nucleosome-induced immunogenicity.

## Discussion

In this study, we demonstrated that extracellular mononucleosomes and oligonucleosomes can be taken up by mammalian cells in the absence of any delivery reagents. After cellular entry, nucleosomes are recognized by the cytoplasmic pattern recognition receptor cGAS, leading to the activation of cGAS. This change finally results in the production of type I interferons and inflammatory cytokines, such as IL-6.

DNA is negatively charged and cannot pass through the negatively charged cellular membrane. One strategy that is used for cellular uptake of DNA is condensing DNA with cationic lipids and polymers, such as lipofectamine, PEI and poly(L-Lys) (PLL), to form cationic liposomes or nanoparticles, which can be taken up by cells via endocytosis^44^. In this context, it is not surprising that nucleosomes can penetrate membranes, given that they are complexes formed by DNA with Lys- and Arg-rich cationic histones^1^. Although several reports have shown that packing plasmid DNA into chromatin can enhance the delivery of plasmid into cells^36,45^, systematic studies on the efficiency and mechanism of cellular uptake of nucleosomes are lacking^46^. In the present study, we demonstrated that both reconstituted mononucleosomes and oligonucleosomes can be taken up by mammalian cells. The 185-nucleosomes and oligonucleosomes showed highly similar cellular permeability, but their cellular uptake was less efficient relative to 145-nucleosomes.

Nanoparticles (NPs) larger than 6 nm can be taken up by HeLa cells via energy-dependent endocytosis, and the uptake of cationic NPs is more efficient than that of zwitterionic and anionic NPs^47^. Nucleosomes are zwitterionic NPs with cylindrical shapes (diameter 11 nm, height 6 nm). The negative charge and positive charge are ascribed to the phosphate linkage of DNA and Lys/Arg residues of histones, respectively. Although the number of negative charges (N_p−_ = 288) is larger than the total number of positive charges (N_(Lys+Arg)+_ = 220) in a NCP (such as the 145-nucleosome), the surface of nucleosomes is positively charged because most Lys and Arg resides are distributed in histone tails that extrude from the NCP, whereas half of the phosphate linkages are buried inside the NCP. The interactions between positively charged histone tails and negatively charged cellular membranes are hypothesized to facilitate the cellular uptake of NCP. We found that removal of histone tails in nucleosomes led to significantly depressed cellular uptake efficiency. In addition, the observation that 185-nucleosomes and oligonucleosomes showed decreased cellular uptake efficiency relative to 145-nucleosomes provides further support for the above hypothesis. In 185-nucleosomes and oligonucleosomes, the free linker DNA that stretches out from the core particle can interact with histone tails through electrostatic interactions and thus decrease the net positive charge on the surface of nucleosomes.

We showed that not only in vitro reconstituted, unmodified nucleosomes but also isolated HeLa nucleosomes were taken up by HeLa cells. In addition, nucleosomes were permeable towards all mammalian cells examined. These results suggest that the cellular entry of nucleosomes is not related to DNA sequences, modifications in DNA and histones, or to cell types; thus, the entry of circulating nucleosomes into living cells could be a common phenomenon in the body. Confocal fluorescence microscopy analysis of the cellular location of nucleosomes (Figs. 2D, 4, Supplementary Figs. S2, S3) revealed that intact nucleosomes can be efficiently delivered into the cytoplasm, where they are directly bound by cGAS. However, the mechanism of cellular uptake remains to be further elucidated in future research.

The *K*_d_ measurements showed that human cGAS exhibits slightly higher affinity for nucleosomes than for the corresponding dsDNA. However, the rates of nucleosome-induced cGAMP synthesis were only approximately 15% of that induced by dsDNA; in other words, the higher binding affinity of nucleosomes does not lead to higher cGAS activation. While this manuscript was under preparation, Zierhut and Funabiki reported that cGAS showed a twofold higher affinity for mononucleosomes than for dsDNA, but nucleosome-bound cGAS exhibited an threefold reduction in the activity^21^, which is in agreement with our findings in this study. It is worth noting that during normal mitosis or upon transient nuclear envelope rupture, exposure of chromatin to cGAS leads to only marginal activation of cGAS^21,48^. In other words, chromatin is considerably less potent in cGAS activation than isolated nucleosomes, which may be observed because condensed packing of nucleosomes in chromatin significantly reduced the accessibility of nucleosomal DNA to cGAS.

**Figure 6:**
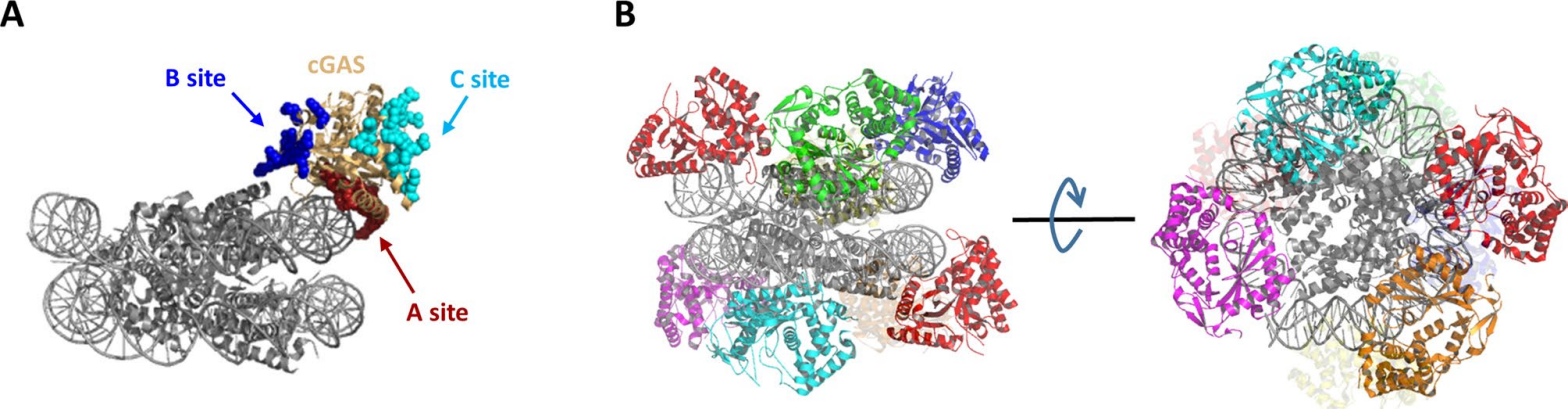
Modelling of the complex formed by binding of cGAS to the 145-nucleosome. (**A**) cGAS binds nucleosomal DNA using the “A site”. (**B**) Multiple cGAS molecules bind to one nucleosome to form a cGAS_n_-nucleosome_1_ complex. The cGAS_n_-nucleosome_1_ model was obtained by docking 145-nucleosome (PDB code: 3LZ0) onto the major dsDNA binding region (“A site”) of cGAS based on the crystal structure of the cGAS_1_-dsDNA_1_ complex (PDB code: 4KB6).

Previous studies have revealed that dsDNA length is the key factor dictating the cGAS activity of cGAMP synthesis^38,43^. cGAS binds a 14 bp dsDNA molecule to form a cGAS_1_-DNA_1_ complex, which shows almost no cGAMP production^37^. Active cGAS_2_-DNA_2_ complexes are formed when mixing 16–20 bp dsDNA with cGAS^38,39^. In this 2:2 complex, one cGAS dimer interacts with two dsDNA molecules, with each cGAS binding the two dsDNA molecules using two separate DNA-binding surfaces (designated “A site” and “B site”, Fig. 6A). For dsDNA molecules longer than 40 bp, multiple cGAS dimers bind along two parallel-aligned dsDNA molecules, forming a cGAS_2n_-DNA_2_ (n ≥ 2) ladder-like complex^40^. The cGAS activity of cGAMP synthesis in different complexes decreases in this order: cGAS_2n_-DNA_2_ > cGAS_2_-DNA_2_ > cGAS_1_-DNA_1_. The variation in cGAS activity is ascribed to different structural rearrangements of cGAS induced by DNA binding in these complexes. In the most potent cGAS_2n_-DNA_2_ ladder-like complex, the long dsDNA adopts a curved conformation, and as a result, interactions between cGAS and dsDNA at the “A site” are reduced. The DNA bending and reduced cGAS-dsDNA interaction play an important role in increasing the selectivity of human cGAS towards long dsDNA^41^.

In this study, we showed that cGAS activation by the nucleosome was due neither to stripping dsDNA from the histone core nor to the free dsDNA of the linker region, which indicated that cGAS must bind nucleosomal DNA on the core region of nucleosomes. Thus, we attempted to manually dock the structure of the 145-nucleosome (PDB code: 3LZ0) onto the dsDNA binding region of cGAS based on the reported dsDNA-cGAS crystal structure (PDB code:4KB6) and found that the structure of the nucleosome precludes the binding of the cGAS dimer; instead, the cGAS monomer can bind to the nucleosome to form a cGAS_n_-nucleosome_1_ complex (Fig. 6). In this model, cGAS binds nucleosomal DNA along the bottom and upper edge of the nucleosome using the “A site” (Fig. 6A), leading to a sandwich structure, with the nucleosome being caught up in the middle by multiple cGAS molecules (Fig. 6B). The nucleosomal DNA bends, and thus, interactions between nucleosomal DNA and cGAS at the “A site” are reduced, which is similar to the situation observed in the cGAS_2n_-DNA_2_ ladder-like complex. Therefore, binding of cGAS to nucleosomal DNA may exert a conformational switch of cGAS towards the active state, which initiates the synthesis of cGAMP. However, the cGAS dimerization that is essential for cGAS activation is not satisfied in this model. Collectively, the potency of the cGAS_n_-nucleosome_1_ in activating cGAS is somewhere between cGAS_2n_-DNA_2_ and the cGAS_1_-nucleosome_1_ complex.

Recently, another DNA binding interface of cGAS, designated “site C” (Fig. 6A), has been identified^49^. Site C is located on the opposite side of sites A and B; thus, site C of cGAS-DNA complexes can further interact with another dsDNA molecule, facilitating multivalent cGAS-DNA complex formation. In the proposed cGAS_n_-nucleosome_1_ model, site C of cGAS is also available for interaction with another nucleosome to form condensed cGAS-nucleosome complexes. This model provides a reasonable explanation for the observation that higher-order cGAS-nucleosome complexes staying on the top of the gel are formed when mixing cGAS with nucleosomes in vitro*.*

Notably, Zierhut and Funabiki recently reported that cGAS binds to nucleosomes via interaction with the acidic patch of H2A-H2B^21^. Alhough we showed that cGAS bound 145-nucleosome and 145-tailless nucleosome with similar affinity (Table 1), this observation could not rule out the possibility of direct interactions between cGAS and histones, given that the tailless nucleosomes remain the intact acidic patch. However, this cGAS–histone interaction, unlike cGAS-DNA interaction, is unlikely to have the capability to activate cGAS. In this context, the cGAS-histone interaction may contribute to the enhanced affinity between nucleosomes and cGAS, whereas the cGAS_n_-nucleosome_1_ model that we proposed represents a reasonable model explaining why nucleosomes can trigger cGAS to synthesize the second messenger cGAMP.

As an extension of the in vitro study, we incubated THP1 cells with different types of nucleosomes and showed that nucleosome entry led to innate immune defence by the production of IFNβ and the inflammatory cytokine IL-6. The colocalization of cGAS with cytoplasmic nucleosomes, together with the significantly inhibited immune responses upon cGAS silencing and knockout, confirmed that nucleosomes trigger the immune response by activating the cGAS-STING pathway. Our results provide an underlying mechanism of nucleosome immunogenicity and help to elucidate many immune-related processes. For instance, dsDNA fragments derived from DNA damage and cell death can gain access to antigen-presenting cells to induce the innate immune response by activating the cGAS-STING pathway. In addition, antitumour immunity induced by DNA damage has recently been found to be dependent on the sensing of tumour-derived DNA in dendritic cells by cGAS^50,51^. However, how cell death- and tumor-derived DNA is delivered to the cytoplasm of antigen-presenting cells is unknown. Our present study suggests that DNA could be released as circulating nucleosomes, which are taken up by antigen-presenting cells to activate the cGAS-STING pathway. In this scenario, circulating nucleosomes play a positive role in antitumour immunity. On the other hand, increased circulating nucleosomes may elicit an improper immune attack by activating cGAS, which provides a reasonable explanation for the association between circulating nucleosomes and systemic autoimmune diseases^2,4,5^. Furthermore, cytoplasmic self-chromatin fragments arising from genome instability can trigger innate immunity by activating the cGAS-STING pathway^15,18^, but the molecular mechanism of how chromatin fragments activate cGAS has not been fully elucidated. Our in vitro studies explicitly demonstrated that mononucleosomes and oligonucleosomes can be recognized and bound by cGAS, leading to cGAS activation. The proposed cGAS_n_-nucleosome_1_ model provides a reasonable explanation for cGAS activation by nucleosomes and chromatin fragments.

In conclusion, we demonstrated that extracellular nucleosomes can be taken up by different types of mammalian cells. The internalization of nucleosomes into immune cells induces the production of type I interferons and inflammatory cytokines. This study revealed the cGAS-dependent immunity of extracellular nucleosomes and highlights the potential roles of circulating nucleosomes in autoimmune diseases, inflammation, and antitumour immunity.

## Methods

### General methods

Expression and purification of histone proteins, as well as refolding and purification of the histone octamer, were described previously^52,53^. Expression and purification of human cGAS was carried out following a reported protocol^42^. Quantification of FAM-labelled dsDNA was carried out using a Amersham Typhoon Gel and Blot Imaging System equipped with ImageQuant Version TL software. The UPLC/MS system is an Acquity UPLC H-Class/Xevo G2 QTof from Waters.

### Preparation of dsDNA

dsDNA molecules were prepared by PCR amplification. The plasmid pGEM-3Z-601^24^ was used as the template for 145-dsDNA and 185-dsDNA, and pD-mNeonGreen-HA300-300 was used for 2,450-dsDNA. After PCR amplification, 145-dsDNA and 185-dsDNA were ethanol precipitated, followed by purification using 8% nondenaturing PAGE. The target bands were excised from the gel, and DNA was eluted overnight using 3 ml of elution buffer (0.2 M NaCl and 1 mM EDTA). The slurry was filtered, followed by ethanol precipitation to provide dsDNA for subsequent studies. Then, 5′-FAM-labelled dsDNA molecules were prepared following the same protocol except that 5′-FAM-labelled forward primers were used. Furthermore, 2,450-dsDNA was prepared using an EasyPure PCR Purification Kit (Transgen Biotech) according to the manufacturer’s instructions.

### Reconstitution of nucleosomes

The 145- and 185-nucleosomes were reconstituted as previously described^27^. Briefly, the 145-/185-dsDNA (100 pmol) and histone octamer (120 pmol) were combined in a dialysis unit to a final volume of 100 μl containing 2 M NaCl. The dialysis unit was placed inside another dialysis bag which was filled with high-salt buffer (2 M NaCl, 10 mM HEPES pH 7.5, 1 mM EDTA, total volume 20 ml). This dialysis bag was then placed into 2 l of low-salt buffer (10 mM HEPES, pH 7.5, 1 mM EDTA) and left for dialysis overnight at 4 °C. Oligonucleosomes were reconstituted following the same procedure except that 20 pmol of 2,450-dsDNA and 336 pmol of histone octamer were used. The reconstitution efficiency was analysed by 5% native PAGE (for 145-nucleosome and 185-nucleosome) or 0.5% agarose electrophoresis (for oligonucleosomes). Both gels were run with 0.2 × TBE buffer.

### Preparation of HeLa nucleosomes

HeLa nucleosomes were prepared as described in previous literature^54^ with minor modifications. HeLa cells (7.0 × 10^7^) were lysed in 2 ml lysis buffer (3 mM MgCl_2_, 0.25 mM sucrose, 0.5% v/v NP40, 0.1 mM PMSF, 1 μM pepstatin A, 1 μM leupeptin, 20 mM HEPES, pH 7.5) using a Tissue Grinder (COYOTE Model:G50). Nuclei, harvested by gentle centrifugation, were broken by the Tissue Grinder in 4 pellet volumes of high-salt buffer (0.65 M NaCl, 1 mM EDTA, 0.34 M sucrose, 1 mM β-mercaptoethanol, 0.5 mM PMSF and 20 mM HEPES, pH 7.5). The mixture was dialyzed overnight at 4 °C against low-salt buffer (0.1 M NaCl, 1 mM EDTA, 1 mM β-mercaptoethanol, 0.5 mM PMSF, and 20 mM HEPES, pH 7.5). CaCl_2_ was added to the obtained sample (final 3 mM) and then allowed to warm to 37 °C. Micrococcal nuclease (final 50 unit/ml) was added, and the mixture was incubated at 37 °C for 5 min, followed by quenching with 0.1 volume of 0.5 M EGTA. While gently vortexing, 2 M NaCl was added dropwise to a final concentration of 0.6 M. Nucleosomes were purified by size exclusion chromatography through a Superdex-200 column and eluted using elution buffer (0.65 M NaCl, 1 mM EDTA, 1 mM β-mercaptoethanol, 0.5 mM PMSF and 20 mM HEPES, pH 7.5). The fractions containing mononucleosomes were combined and dialyzed against dialysis buffer (10 mM HEPES, pH 7.5, 100 mM NaCl, 1 mM EDTA, 0.1 mM PMSF) at 4 °C overnight. Nucleosomes were concentrated to 3 mg/ml using Centriprep-10 concentrators (Amicon) and stored on ice.

### FAM labelling of HeLa nucleosomes

First, 0.4 μl of 5(6)-carboxyfluorescein *N*-hydroxysuccinimide ester (FAM-NHS) (prepared by dissolving 1 mg of FAM-NHS in 200 µl DMSO) was added to a solution of purified HeLa nucleosomes (100 ng DNA/μl, 400 μl). The reaction mixture was incubated at 37 °C for 1 h. Excess FAM-NHS was removed by dialysis (10 mM HEPES, pH 7.5, 20 mM NaCl, 1 mM EDTA, 0.1 mM PMSF) overnight at 4 °C.

### Preparation of Cy5-labelled histone H3 and histone octamers

The plasmid used for expression of histone mutant H3-V35C-C110A was generated using the QuikChange site-directed mutagenesis kit (Tiangen) and the histone mutant was expressed in *E. coli* strain BL21 (DE3) as previously described^53^. To prepare Cy5-conjugated H3, Cy5-maleimide (10 μl, 200 mM in DMSO) was added to a solution of H3-V35C-C110A (2 ml, 50–100 μM) in HEPES buffer (50 mM, pH 7.5). The reaction mixture was incubated at 37 °C for 2 h. Excess Cy5-maleimide and salts were removed by PD Minitrap G-25 column (GE Healthcare) according to the spin protocol. Cy5-H3 was mixed with histone H4, H2A and H2B to prepare Cy5-labelled histone octamers using a previously described protocol^52^.

### Cell culture and nucleosome treatment

Cell lines, including HeLa, HEK293T, HepG2, LO2, BMDM, L929 and L929-*Cgas*^*−/−*^, were cultured in DMEM (Gibco) supplemented with 10% foetal bovine serum (FBS), 50 U/ml penicillin and 50 μg/ml streptomycin at 37 °C in 5% CO_2_. THP-1 cells were cultured in RPMI-1640 (Gibco) containing 10% FBS, 50 U/ml penicillin and 50 μg/ml streptomycin, 2 mM l-glutamine and 50 μM of β-mercaptoethanol at 37 °C in 5% CO_2_. For nucleosome treatment, cells were seeded into 48-well (9.0 × 10^4^) or 24-well plates (3.0 × 10^5^). After 12 h incubation (approximately 85% confluence), the medium was changed to Opti-MEM. Then, nucleosomes or dsDNA of the indicated concentrations were added to the medium and incubation was continued for periods as mentioned in the text. dsDNA transfection by Lipofectamine 2000 (Invitrogen) was carried out according to the manufacturer’s instructions. For RT-qPCR and ELISA analyses, which required a long incubation time (24 h), no Opti-MEM was applied. Instead, normal medium with 10% FBS was used in the whole process.

### Confocal microscopy

Cells grown overnight on glass coverslips were treated with fluorescently labelled nucleosomes or dsDNA for varying periods. After washing with PBS twice, cells were fixed in 4% paraformaldehyde (PFA) for 20 min at room temperature. Then, the fixed cells were washed in PBS and stained with DAPI, and coverslips were mounted onto clean glass slides. Image capture was performed using an OLYMPUS FV1000S-IX81 laser scanning confocal microscope.

### Flow cytometry

Cells were seeded into 48-well plates at a density of 9.0 × 10^4^ cells/well. After incubation overnight and medium change, fluorescently labelled nucleosomes or dsDNA of the indicated concentrations were added, and incubation was continued for periods as mentioned in the text. The cells were harvested and washed with PBS and suspended in PBS. Then, the samples were immediately analysed by flow cytometry (BD, USA).

### Electrophoretic mobility shift assay (EMSA) of binding of cGAS to nucleosomes and dsDNA

FAM-labelled nucleosome/dsDNA (0.8 pmol) or oligonucleosomes/2,450-dsDNA (0.1 pmol) were mixed with different amounts of cGAS in binding buffer (20 mM Tris–HCl, pH 7.6, 60 mM NaCl, and 5 mM MgCl_2_, total volume 10 μl). After incubation on ice for 30 min, the DNA–protein mixture was loaded onto a 1.5% agarose gel and electrophoresed for 25 min at 150 V in 0.2 × TBE.

### In vitro assay of cGAS activity

Human cGAS was incubated in a 20 μl reaction system mainly containing 2 μM cGAS protein, 2.5 mM ATP, 2.5 mM GTP, 5 mM MgCl_2_, and dsDNA or nucleosomes (80 ng/μl DNA). After incubation at 37 °C for 3 h, the mixture was heated at 95 °C for 5 min and centrifuged at 16,000×*g* for 20 min. Then, the supernatant was analysed by UPLC-MS/MS.

### Immunofluorescence

Cells were seeded into 48 well plates with a density of 3.0 × 10^4^ cells/well. After incubation overnight and medium change, FAM-labelled or dual-labelled nucleosomes were added to the medium (final 20 nM) and incubation was continued for 12 h. After washing with PBS twice, cells were fixed in 4% paraformaldehyde for 20 min at room temperature, permeabilized for 10 min in 0.3% (v/v) Triton X-100 and blocked with 5% BSA for 30 min at room temperature. Cells were incubated with rabbit anti-EEA1 antibody (Abcam, Cat #109110, diluted 1:500) or anti-MB21D1(cGAS) rabbit anti-mouse antibody (ABclonal, Cat #8335, diluted 1:100) overnight at 4 °C. After washing with PBS twice, cells were incubated with the secondary antibody, Cy3 goat anti-rabbit IgG (Abclonal, Cat #AS007, diluted 1:100) for 1 h, and then, nuclei were stained with DAPI for 7 min. Image capture was performed using an OLYMPUS FV1000S-IX81 laser scanning confocal microscope.

### Quantitative real-time PCR (RT-qPCR)

After incubation with nucleosomes for the indicated periods, cells were harvested. Total RNA was extracted from cells using RNAiso Plus (TaKaRa, Ohtsu, Japan). First-strand cDNA was synthesized by TransScript First-Strand cDNA Synthesis SuperMix (Transgen Biotech). RT-qPCR was performed using a Bio-Rad sequence detection system according to the manufacturer's instructions using TransStart Top Green qPCR SuperMix (Transgen Biotech). Experiments were conducted in duplicate in three independent assays. Relative transcriptional folds were calculated as 2^-∆∆Ct^. ACTIN was used as an internal control for normalization. Primers for RT-qPCR analysis are shown in Supplementary Table S1.

### ELISA

THP1 cells were seeded into 24 well plates at a density of 3.0 × 10^5^ cells/well with RPMI-1640 medium. After incubation overnight , nucleosomes were added to the medium (final 20 nM), and incubation was continued for 24 h. A brief centrifuge at 2,000×*g* was applied to remove dead cells and cell debris, and the supernatants were subjected to ELISA analysis. ELISA was performed following the manufacturer’s instructions of the Human IFN-β ELISA Kit (Elabscience) and IL-6 ELISA Kit (Abclonal).

### RNAi

The siRNAs were purchased from RiboBio (Guangzhou, China). The sequences of the siRNA oligos were as follows. cGAS-1: GAAGAAACAUGGCGGCUAU. cGAS-2: AGAGAAAUGUUGCAGGAAA^55^. siRNAs were transfected into THP-1 cells using Lipofectamine 2000 according to the manufacturer’s instructions. After incubation for 24 h, cells were harvested, washed with PBS twice, and seeded into 24-well plates with RPMI-1640 medium. Nucleosomes were added to the medium (final 20 nM), and incubation was continued for 24 h. Cells and supernatants were collected and subjected to RT-qPCR and ELISA analysis, respectively.

### Western blotting (WB)

L929 and L929-Cgas^−/−^ cells were lysed with RIPA buffer (Solarbio, China). The whole-cell lysates were collected after centrifugation at 1,000×*g* for 10 min at 4 °C, subjected to 10% SDS-PAGE, and transferred to immunoblot PVDF membrane (Millipore). The membrane was probed with rabbit anti-MB21D1(cGAS) antibody (ABclonal, Cat #A8335, diluted 1:1,000) or beta actin antibody rabbit polyclonal (Proteintech, Cat #20536–1-AP, diluted 1:2,500), and then with horseradish-peroxidase-conjugated secondary antibody directed against rabbit IgG (Proteintech, Cat #SA00001-2, diluted 1:4,000). After incubation with Western Lightning BeyoECL Plus (Beyotime, China), the membrane was visualized with on the Tanon-5500 Chemiluminescent Imaging System (Tanon Science and Technology, Shanghai, China).

### Statistical analysis

We performed statistical analysis by Student’s *t*-test. Significance levels of p > 0.05, 0.05 > p > 0.01, 0.01 > p > 0.001, 0.001 > p are denoted in graphs by ns, a single, double, or triple asterisk, respectively.

## Supplementary information

Supplementary Information

## References

[1] McGinty RK, Tan S (2015). Nucleosome structure and function. Chem. Rev..

[2] Marsman G, Zeerleder S, Luken BM (2016). Extracellular histones, cell-free DNA, or nucleosomes: Differences in immunostimulation. Cell Death Dis..

[3] Holdenrieder S (2001). Circulating nucleosomes in serum. Ann. N. Y. Acad. Sci..

[4] Pisetsky DS (2012). The origin and properties of extracellular DNA: From PAMP to DAMP. Clin. Immunol..

[5] Schwarzenbach H, Hoon DSB, Pantel K (2011). Cell-free nucleic acids as biomarkers in cancer patients. Nat. Rev. Cancer.

[6] Ronnefarth VM (2006). TLR2/TLR4-independent neutrophil activation and recruitment upon endocytosis of nucleosomes reveals a new pathway of innate immunity in systemic lupus erythematosus. J. Immunol..

[7] Roers A, Hiller B, Hornung V (2016). Recognition of endogenous nucleic acids by the innate immune system. Immunity.

[8] Hu M-M, Shu H-B (2018). Transcriptional control of developmental cell behaviors. Annu. Rev. Cell Dev. Biol..

[9] Ablasser A, Chen ZJJ (2019). cGAS in action: Expanding roles in immunity and inflammation. Science.

[10] Sun L, Wu J, Du F, Chen X, Chen ZJ (2013). Cyclic GMP-AMP synthase is a cytosolic DNA sensor that activates the type I interferon pathway. Science.

[11] Wu JX (2013). Cyclic GMP-AMP is an endogenous second messenger in innate immune signaling by cytosolic DNA. Science.

[12] Ablasser A (2013). cGAS produces a 2'-5'-linked cyclic dinucleotide second messenger that activates STING. Nature.

[13] Galluzzi L, Vanpouille-Box C, Bakhoum SF, Demaria S (2018). SnapShot: cGAS-STING signaling. Cell.

[14] Barber GN (2015). STING: Infection, inflammation and cancer. Nat. Rev. Immunol..

[15] Dou Z (2017). Cytoplasmic chromatin triggers inflammation in senescence and cancer. Nature.

[16] Gluck S (2017). Innate immune sensing of cytosolic chromatin fragments through cGAS promotes senescence. Nat. Cell Biol..

[17] Harding SM (2017). Mitotic progression following DNA damage enables pattern recognition within micronuclei. Nature.

[18] Mackenzie KJ (2017). cGAS surveillance of micronuclei links genome instability to innate immunity. Nature.

[19] Bakhoum SF (2018). Chromosomal instability drives metastasis through a cytosolic DNA response. Nature.

[20] Lahaye X (2018). NONO detects the nuclear HIV capsid to promote cGAS-mediated innate immune activation. Cell.

[21] Zierhut C (2019). The cytoplasmic DNA sensor cGAS promotes mitotic cell death. Cell.

[22] Jahr S (2001). DNA fragments in the blood plasma of cancer patients: Quantitations and evidence for their origin from apoptotic and necrotic cells. Cancer Res..

[23] Nagata S (2000). Apoptotic DNA fragmentation. Exp. Cell Res..

[24] Lowary PT, Widom J (1998). New DNA sequence rules for high affinity binding to histone octamer and sequence-directed nucleosome positioning. J. Mol. Biol..

[25] Vasudevan D, Chua EYD, Davey CA (2010). Crystal structures of nucleosome core particles containing the '601' strong positioning sequence. J. Mol. Biol..

[26] Zhou CZ, Greenberg MM (2012). Histone-catalyzed cleavage of nucleosomal DNA containing 2-deoxyribonolactone. J. Am. Chem. Soc..

[27] Li F (2017). 5-Formylcytosine yields DNA–protein cross-links in nucleosome core particles. J. Am. Chem. Soc..

[28] Bai J, Zhang Y, Xi Z, Greenberg MM, Zhou C (2018). Oxidation of 8-oxo-7,8-dihydro-2′-deoxyguanosine leads to substantial DNA-histone cross-links within nucleosome core particles. Chem. Res. Toxicol..

[29] Ren M, Cheng Y, Duan Q, Zhou C (2019). Transesterification reaction and the repair of embedded ribonucleotides in DNA are suppressed upon the assembly of DNA into nucleosome core particles. Chem. Res. Toxicol..

[30] Ren M, Bai J, Xi Z, Zhou C (2019). DNA damage in nucleosomes. Sci. China Chem..

[31] Shang M, Ren M, Zhou C (2019). Nitrogen mustard induces formation of DNA–histone cross-links in nucleosome core particles. Chem. Res. Toxicol..

[32] Weng LW, Greenberg MM (2015). Rapid histone-catalyzed DNA lesion excision and accompanying protein modification in nucleosomes and nucleosome core particles. J. Am. Chem. Soc..

[33] Song F (2014). Cryo-EM study of the chromatin fiber reveals a double helix twisted by tetranucleosomal units. Science.

[34] Li G, Levitus M, Bustamante C, Widom J (2005). Rapid spontaneous accessibility of nucleosomal DNA. Nat. Struct. Mol. Biol..

[35] Rosenbluh J (2005). Translocation of histone proteins across lipid bilayers and mycoplasma membranes. J. Mol. Biol..

[36] Wagstaff KM, Fan JY, De Jesus MA, Tremethick DJ, Jans DA (2008). Efficient gene delivery using reconstituted chromatin enhanced for nuclear targeting. FASEB J..

[37] Civril F (2013). Structural mechanism of cytosolic DNA sensing by cGAS. Nature.

[38] Li X (2013). Cyclic GMP-AMP synthase is activated by double-stranded DNA-induced oligomerization. Immunity.

[39] Zhang X (2014). The cytosolic DNA sensor cGAS forms an oligomeric complex with DNA and undergoes switch-like conformational changes in the activation loop. Cell Rep..

[40] Andreeva L (2017). cGAS senses long and HMGB/TFAM-bound U-turn DNA by forming protein–DNA ladders. Nature.

[41] Zhou W (2018). Structure of the human cGAS-DNA complex reveals enhanced control of immune surveillance. Cell.

[42] Tao J (2017). Nonspecific DNA binding of cGAS N terminus promotes cGAS activation. J. Immunol..

[43] Luecke S (2017). cGAS is activated by DNA in a length-dependent manner. EMBO Rep..

[44] Mintzer MA, Simanek EE (2009). Nonviral vectors for gene delivery. Chem. Rev..

[45] Kamiya H (2013). Enhanced transgene expression from chromatinized plasmid DNA in mouse liver. Int. J. Pharm..

[46] Kontouzov S (1996). Binding of nucleosomes to a cell surface receptor: Redistribution and endocytosis in the presence of lupus antibodies. Eur. J. Immunol..

[47] Jiang Y (2015). The interplay of size and surface functionality on the cellular uptake of sub-10 nm gold nanoparticles. ACS Nano.

[48] Gentili M (2019). The N-terminal domain of cGAS determines preferential association with centromeric DNA and innate immune activation in the nucleus. Cell Rep..

[49] Xie W (2019). Human cGAS catalytic domain has an additional DNA-binding interface that enhances enzymatic activity and liquid-phase condensation. Proc. Natl. Acad. Sci. USA.

[50] Deng L (2014). STING-dependent cytosolic DNA sensing promotes radiation-induced type I interferon-dependent antitumor immunity in immunogenic tumors. Immunity.

[51] Woo S-R (2014). STING-dependent cytosolic DNA sensing mediates innate immune recognition of immunogenic tumors. Immunity.

[52] Dyer PN (2004). Reconstitution of nucleosome core particles from recombinant histones and DNA. Methods Enzymol..

[53] Sczepanski JT, Zhou CZ, Greenberg MM (2013). Nucleosome core particle-catalyzed strand scission at abasic sites. Biochemistry.

[54] Schnitzler GR (2001). Isolation of histones and nucleosome cores from mammalian cells. Curr. Protoc. Mol. Biol..

[55] Herzner A-M (2015). Sequence-specific activation of the DNA sensor cGAS by Y-form DNA structures as found in primary HIV-1 cDNA. Nat. Immunol..

