# Supplementary material for: Cellular uptake of extracellular nucleosomes induces innate immune responses by binding and activating cGMP-AMP synthase (cGAS)
Source: Sci Rep. 2020 Sep 21;10:15385. doi: 10.1038/s41598-020-72393-w (PMC7505961; doi:10.1038/s41598-020-72393-w)
Supplement: Supplementary file 1 — Supplementary Information [file 41598_2020_72393_MOESM1_ESM.pdf]

Supplementary Information

Cellular uptake of extracellular nucleosomes induces innate immune responses by binding and activating cGMP-AMP synthase (cGAS)

Huawei Wang[1], Chuanlong Zang[1], Mengtian Ren[1], Mengdi Shang[1], Zhenghua Wang[1], Xuemei Peng[2], Qiangzhe Zhang[2], Xin Wen[1], Zhen Xi[1], Chuanzheng Zhou[1,*]

[1] State Key Laboratory of Elemento-Organic Chemistry and Department of Chemical Biology, College of Chemistry, Nankai University, Tianjin 300071, China

[2] State Key Laboratory of Medicinal Chemical Biology and College of Pharmacy, Tianjin Key Laboratory of Molecular Drug Research, Nankai University, Tianjin 300071, China

* To whom correspondence should be addressed. Email: chuanzheng.zhou@nankai.edu.cn

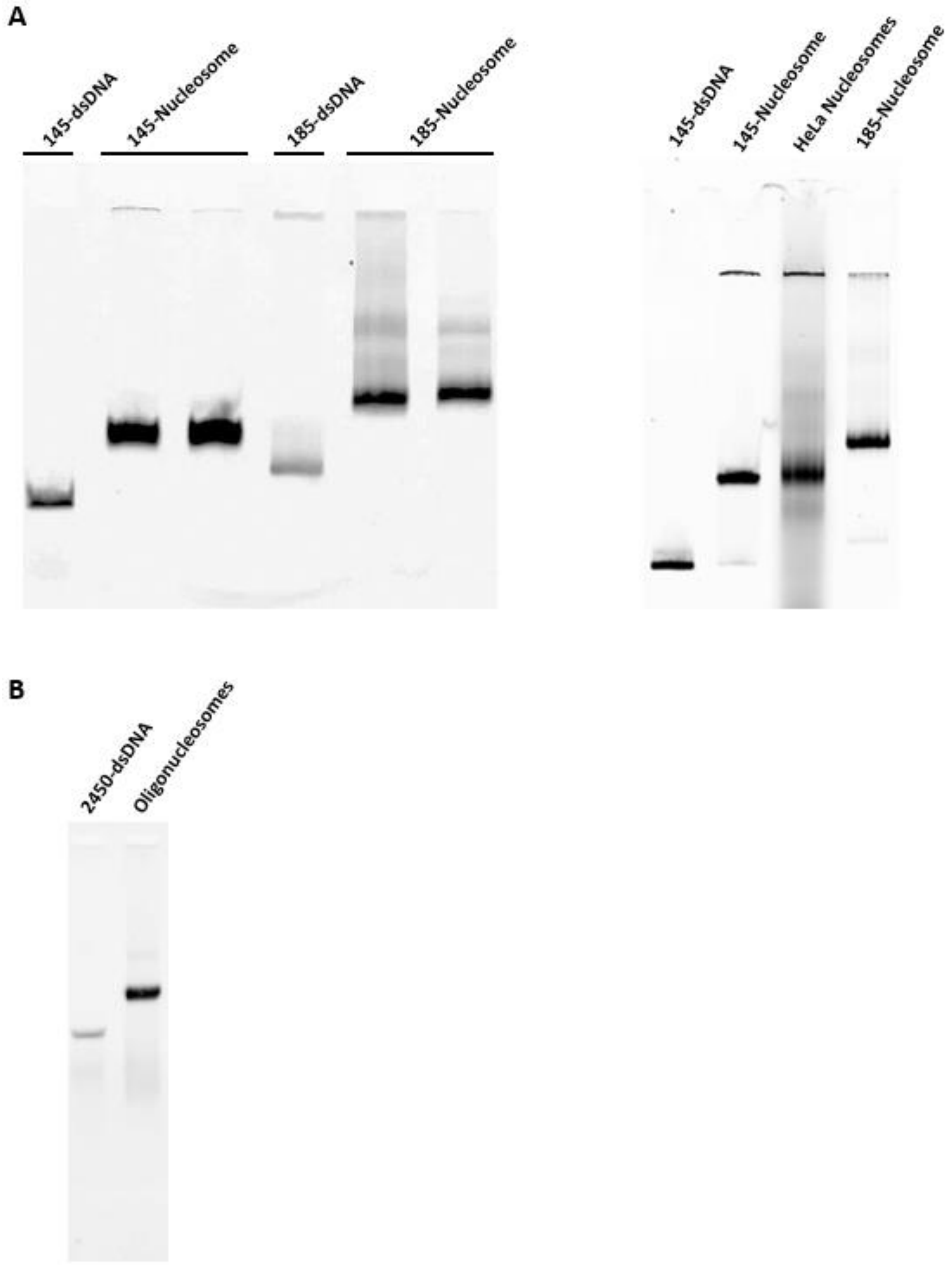

Supplementary Figure S1. Nondenaturing gel analyses of the prepared nucleosomes. (A) 5% native gel analysis of 145-nucleosome, 185-nucleosome and isolated HeLa nucleosomes. (B) 0.5% Agarose gel analysis of oligonucleosomes. The gels were visualized based on fluorescence imaging of FAM moieties that are tagged either on DNA (for 145-, 185- and oligonucleosome) or on histones (for HeLa nucleosomes).

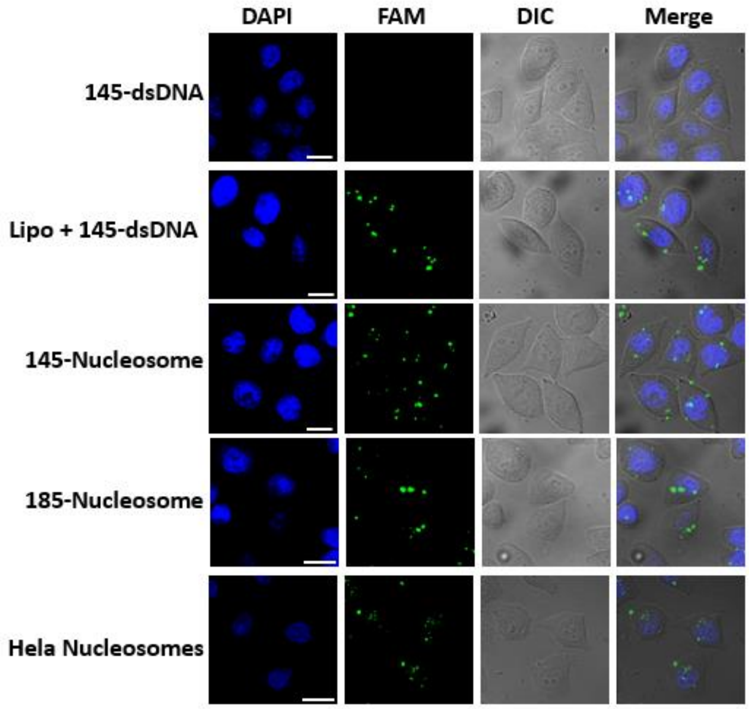

Supplementary Figure S2. Confocal fluorescence microscopy analysis of the cellular location of nucleosomes. HeLa cells were cultured in opti-MEM medium containing FAM-labelled 145-dsDNA or nucleosomes (20 nM) for 6 hours. After fixation, the cells were visualized using confocal fluorescence microscopy. The FAM channel was obtained by excitation at 488 nm and emission at 526 nm. Scale bar, 20 μm.

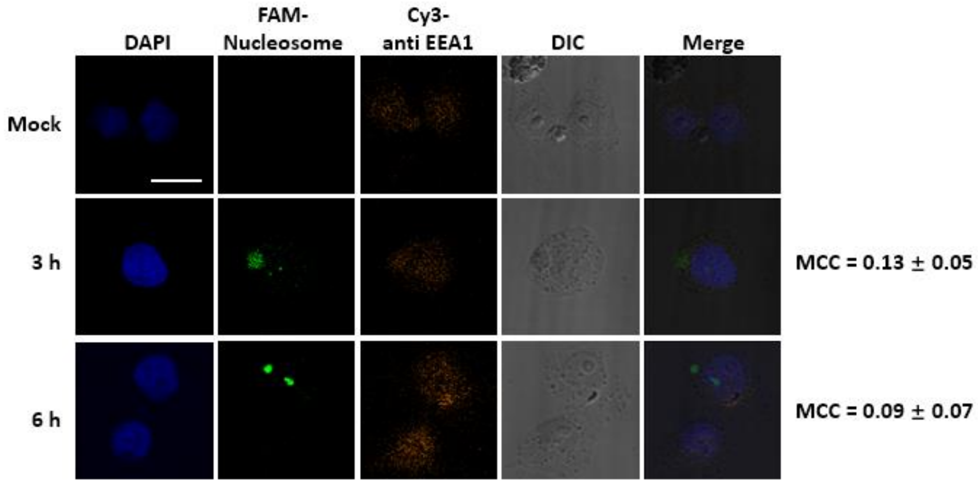

Supplementary Figure S3. Confocal fluorescence microscopy analysis of the cellular location of FAM-labelled 145-nucleosomes and early endosome marker EEA1 in HeLa cells. MCC (Manders' colocalization coefficients) is the ratio of FAM signal that overlaps with Cy3 to the total signal of FAM. Scale bar, 20 μm.

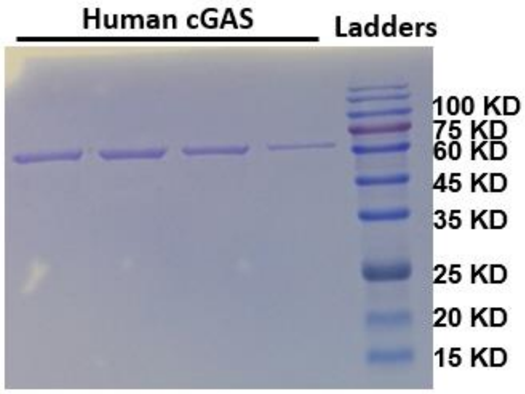

Supplementary Figure S4. A 10% SDS PAGE gel showing the purity of expressed human cGAS.

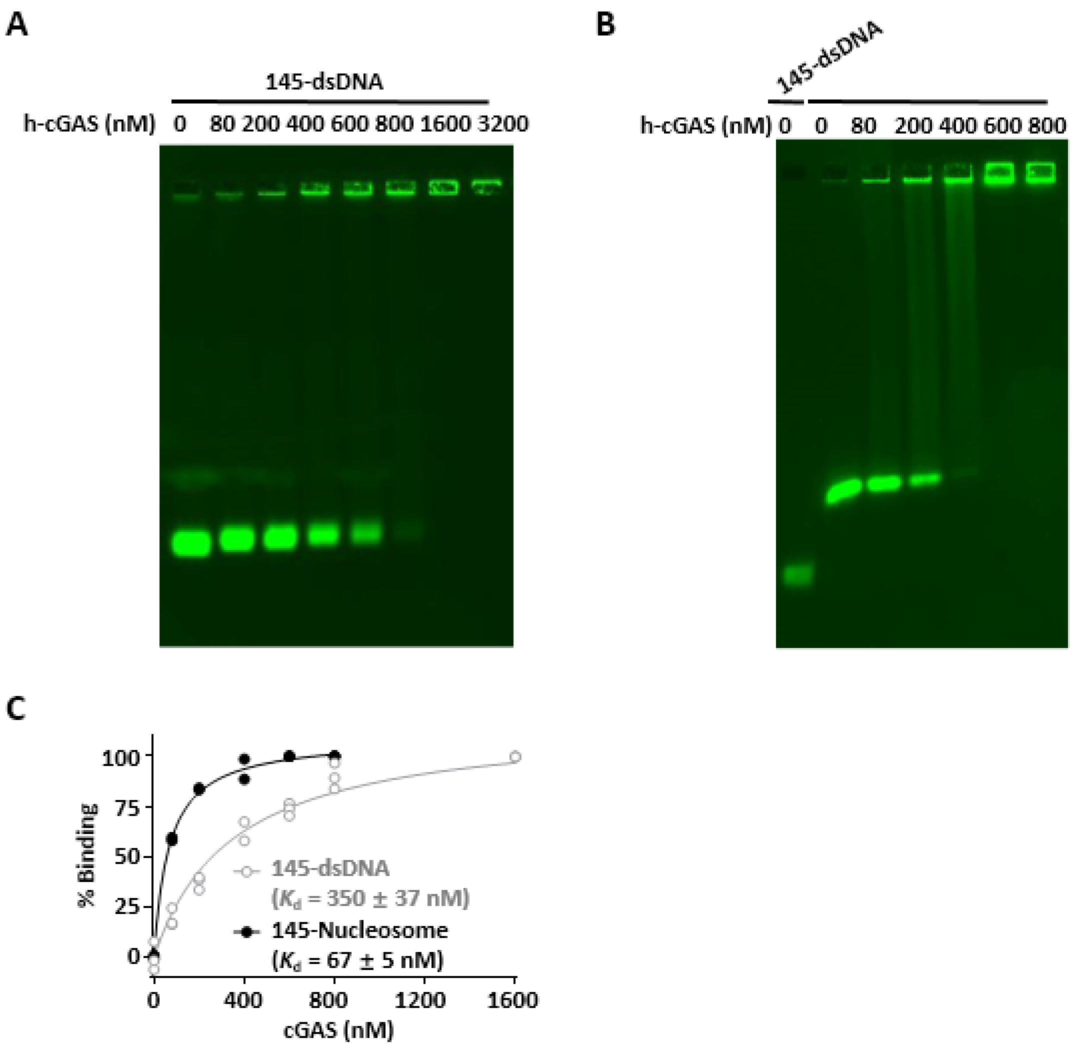

Supplementary Figure S5. Binding of 145-dsDNA and 145-nucleosomes by human cGAS. (**A**) Agarose gel analysis of binding of cGAS to 5’-FAM-labelled 145-dsDNA. (**B**) Agarose gel analysis of binding of cGAS to 5’-FAM-labelled 145-nucleosomes. 145-dsDNA or 145-Nucleosomes (80 nM) were mixed with a variety of concentrations of cGAS in Tris-HCl buffer (20 mM, pH 7.6, 60 mM NaCl, 5 mM $MgCl_2$) in a total of 10 µl. After incubation on ice for 30 min, the mixtures were analysed by 1.5% agarose gel. The gels were visualized by fluorescence imaging with excitation at 488 nm and emission at 526 nm. (C) Plot of fractions of binding DNA or nucleosomes versus the concentration of human cGAS, from which *K*d values were derived. Error bars indicate the mean ± standard deviation of at three independent experiments.

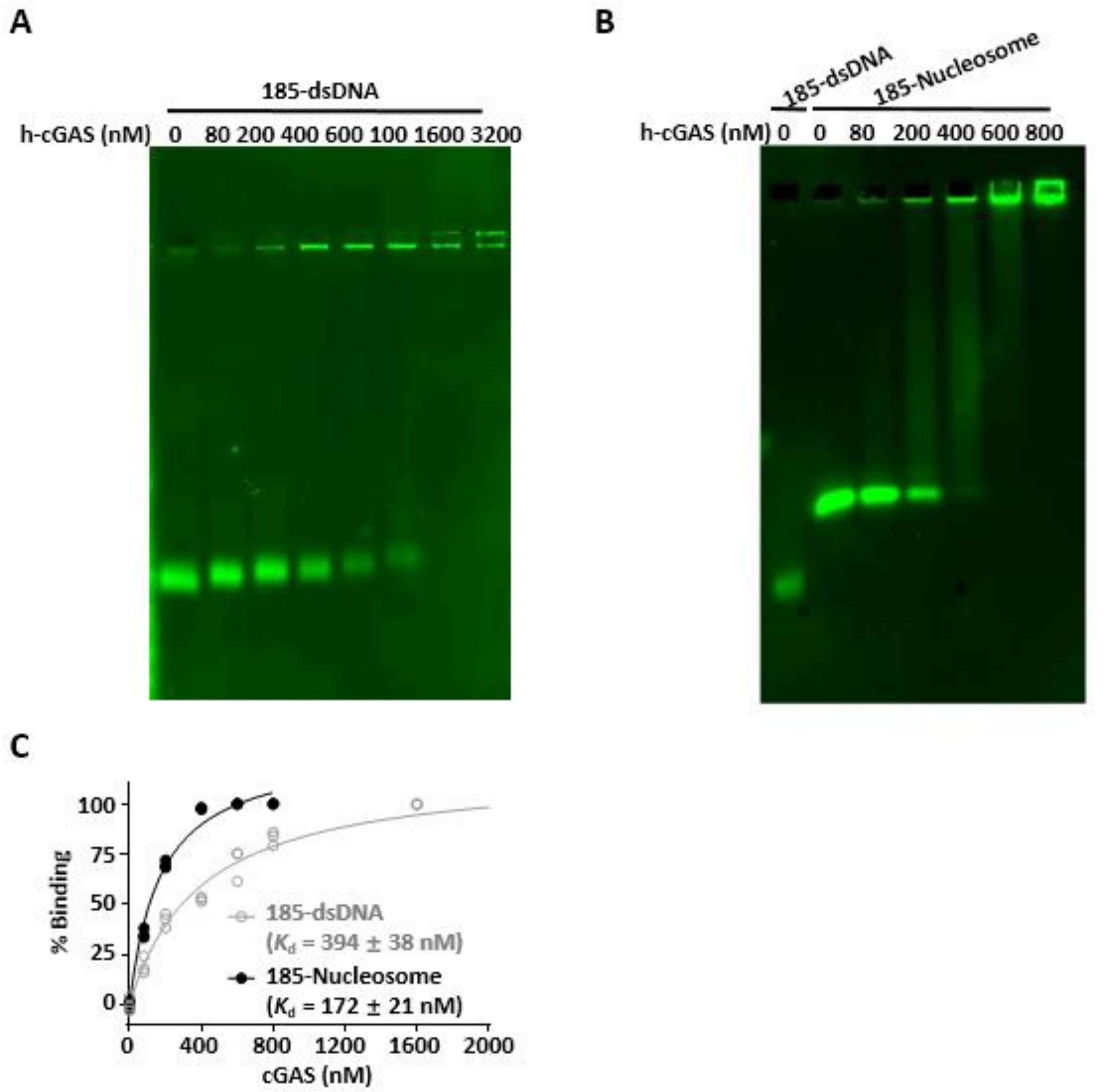

Supplementary Figure S6. Binding of 185-dsDNA and 185-nucleosomes by human cGAS. (**A**) Agarose gel analysis of binding of cGAS to 5’-FAM-labelled 185-dsDNA. (**B**) Agarose gel analysis of binding of cGAS to 5’-FAM-labelled 185-nucleosomes. 185-dsDNA or 185-Nucleosomes (80 nM) were mixed with a variety of concentrations of cGAS in Tris-HCl buffer (20 mM, pH 7.6, 60 mM NaCl, 5 mM $MgCl_2$) in a total of 10 µl. After incubation on ice for 30 min, the mixtures were analysed by 1.5% agarose gel. The gels were visualized by fluorescence imaging with excitation at 488 nm and emission at 526 nm. (C) Plot of fractions of binding DNA or nucleosomes versus the concentration of human cGAS, from which *K*d values were derived. Error bars indicate the mean ± standard deviation of three independent experiments.

.

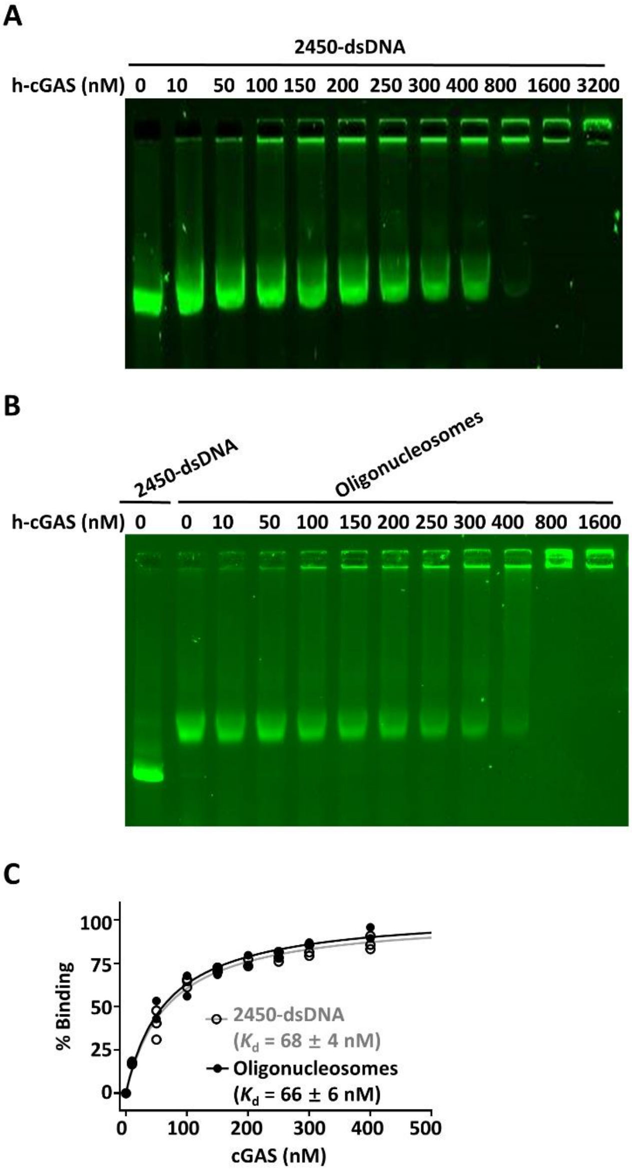

Supplementary Figure S7. Binding of 2450-dsDNA and oligonucleosomes by human cGAS. (**A**) Agarose gel analysis of binding of cGAS to 5'-FAM-labelled 2450-dsDNA. (**B**) Agarose gel analysis of binding of cGAS to 5'-FAM-labelled oligonucleosomes. 2450-dsDNA or oligonulceosomes (10 nM) were mixed with a variety of concentrations of cGAS in Tris-HCl buffer (20 mM, pH 7.6, 60 mM NaCl, 5 mM $MgCl_2$) in a total of 10 µl. After incubation on ice for 30 min, the mixtures were analysed by 1.5% agarose gel. The gels were visualized by fluorescence imaging with excitation at 488 nm and emission at 526 nm. (C) Plot of fractions of binding DNA or nucleosomes versus the concentration of human cGAS, from which *K*d values were derived. Error bars indicate the mean ± standard deviation of three independent experiments.

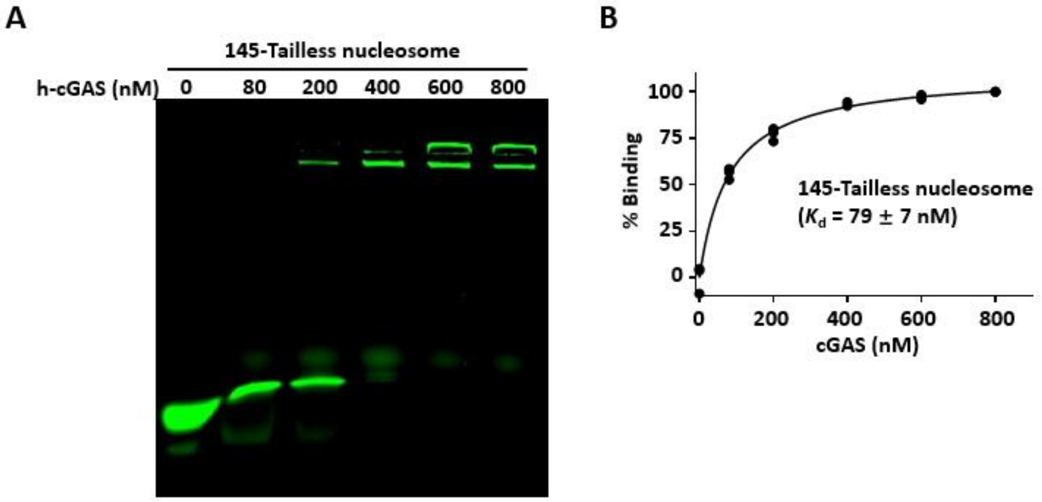

Supplementary Figure S8. Binding 145-tailless nucleosomes by human cGAS. (**A**) Agarose gel analysis of binding of cGAS to 5'-FAM-labelled 145-tailless nucleosomes. 145-tailless nucleosomes (80 nM) were mixed with a variety of concentrations of cGAS in Tris-HCl buffer (20 mM, pH 7.6, 60 mM NaCl, 5 mM $MgCl_2$) in a total of 10 µl. After incubation on ice for 30 min, the mixtures were analysed by 1.5% agarose gel. The gels were visualized by fluorescence imaging with excitation at 488 nm and emission at 526 nm. (**B**) Plot of fractions of binding 145-tailless nucleosomes versus the concentration of human cGAS, from which *K*d values were derived. Error bars indicate the mean ± standard deviation of three independent experiments.

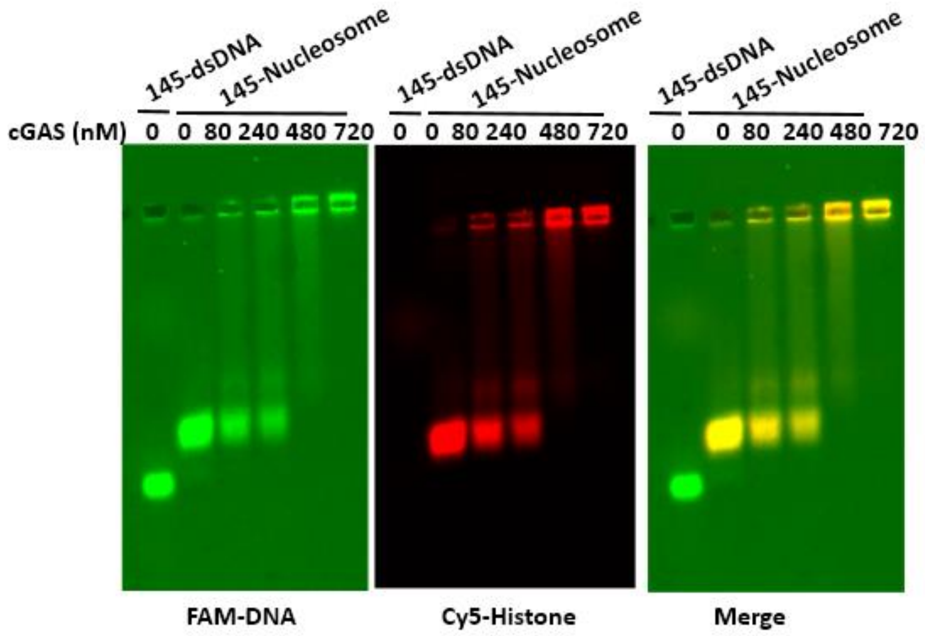

Supplementary Figure S9. Agarose gel analyses of the binding of cGAS to 5'-FAM/ Cy5-H3 dual-labelled 145-nucleosomes. Nucleosomes (80 nM) were mixed with a variety of concentrations of cGAS in Tris-HCl buffer (20 mM, pH 7.6, 60 mM NaCl, 5 mM $MgCl_2$), for a total of 10 µl. After incubation on ice for 30 min, the mixtures were analyzed by 1.5% agarose gel.

A

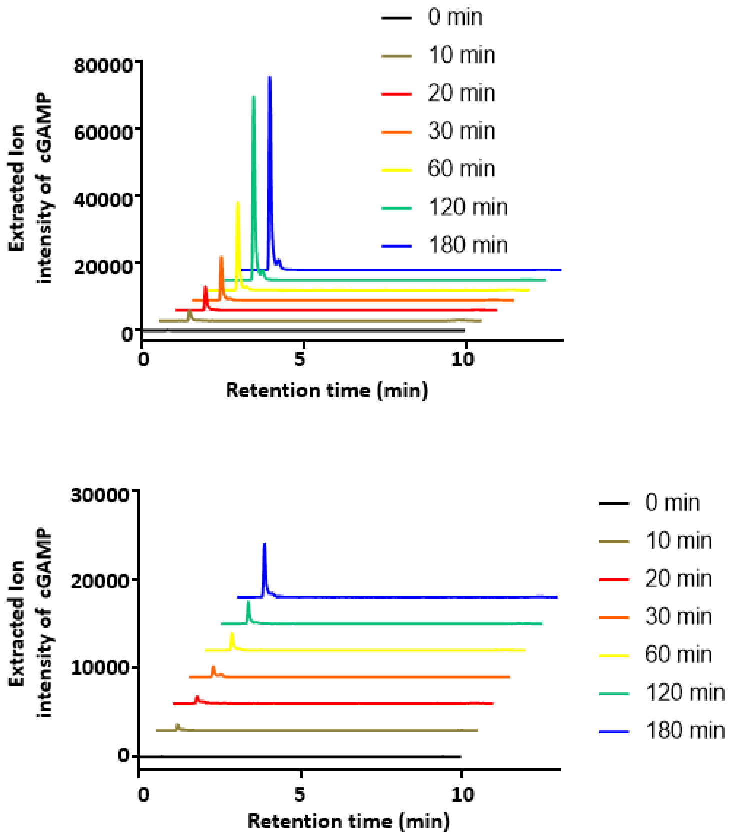

Supplementary Figure S10. UPLC-MS EIC (extracted ion chromatogram) curves for detecting cGAMP generation upon incubation of cGAS with 145-dsDNA and 145-nucleosomes. (**A**) 145-dsDNA. (**B**) 145-Nucleosomes. Nucleosomes or dsDNA (80 ng DNA/μL) were mixed with human cGAS (2 μM) in a Tris-HCl buffer (20 mM, pH 7.6, 60 mM NaCl, 2.5 mM ATP, 2.5 mM GTP, 5 mM $MgCl_2$), incubation at 37 °C. Aliquots were taken and analysed by UPLC-MS.

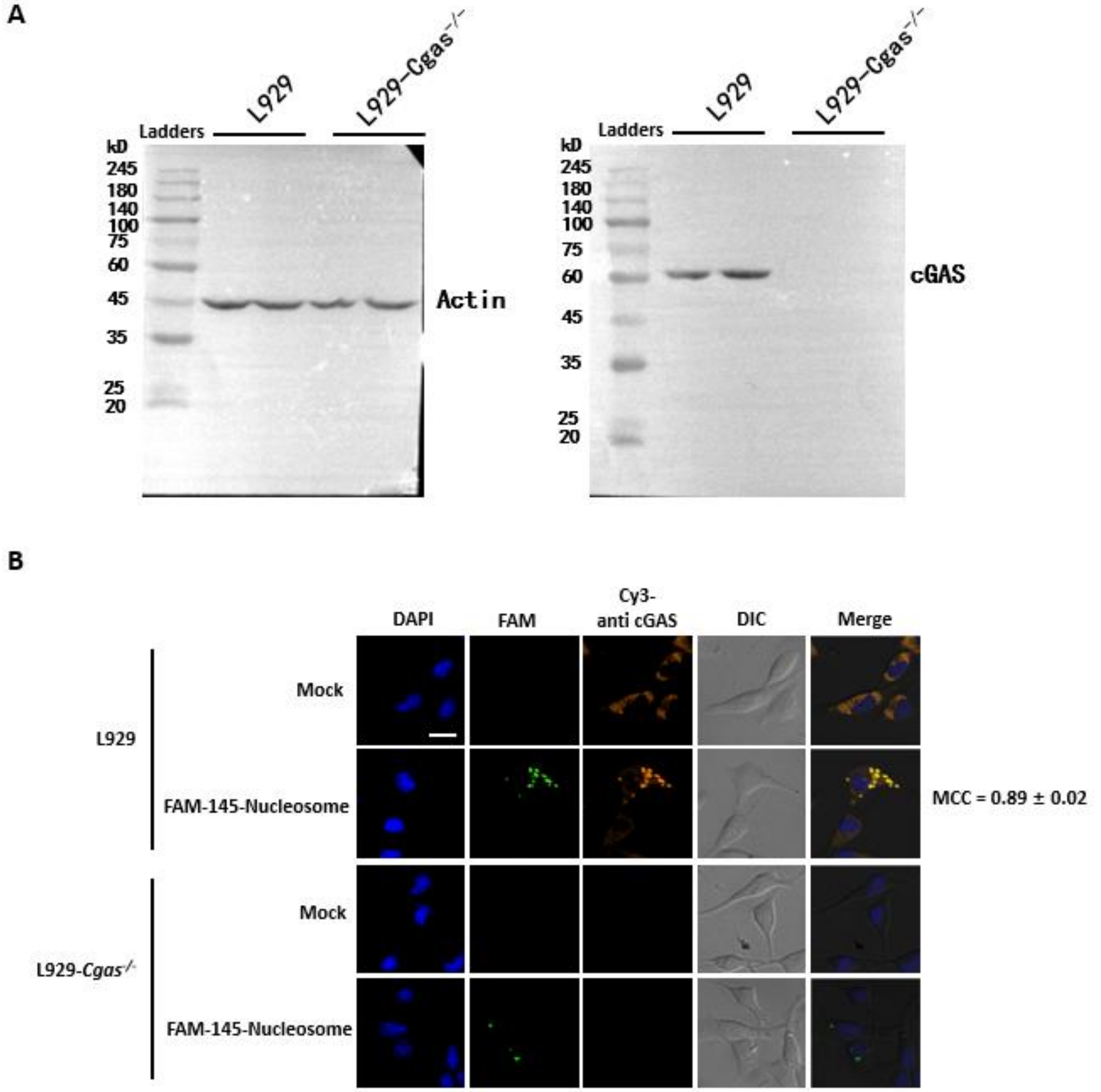

Supplementary Figure S11. Immunoanalysis of cGAS in L929-WT cells and cGAS knockout L929 cells (L929-*Cgas*$^{-/-}$) using anti-cGAS antibody. **(A)** WB showing the cGAS expression in L929-WT cells and L929-*Cgas*$^{-/-}$ cells. **(B)** Confocal fluorescence microscopy analyses of the cellular location of nucleosomes and cGAS in L929-WT and L929-*Cgas*$^{-/-}$cells. Representative images showing the cellular location of nucleosomes and cGAS after incubation for 12 h. Cy3 labeled secondary antibody was used for the immunofluorescence of cGAS. Scale bar, 20 μm.

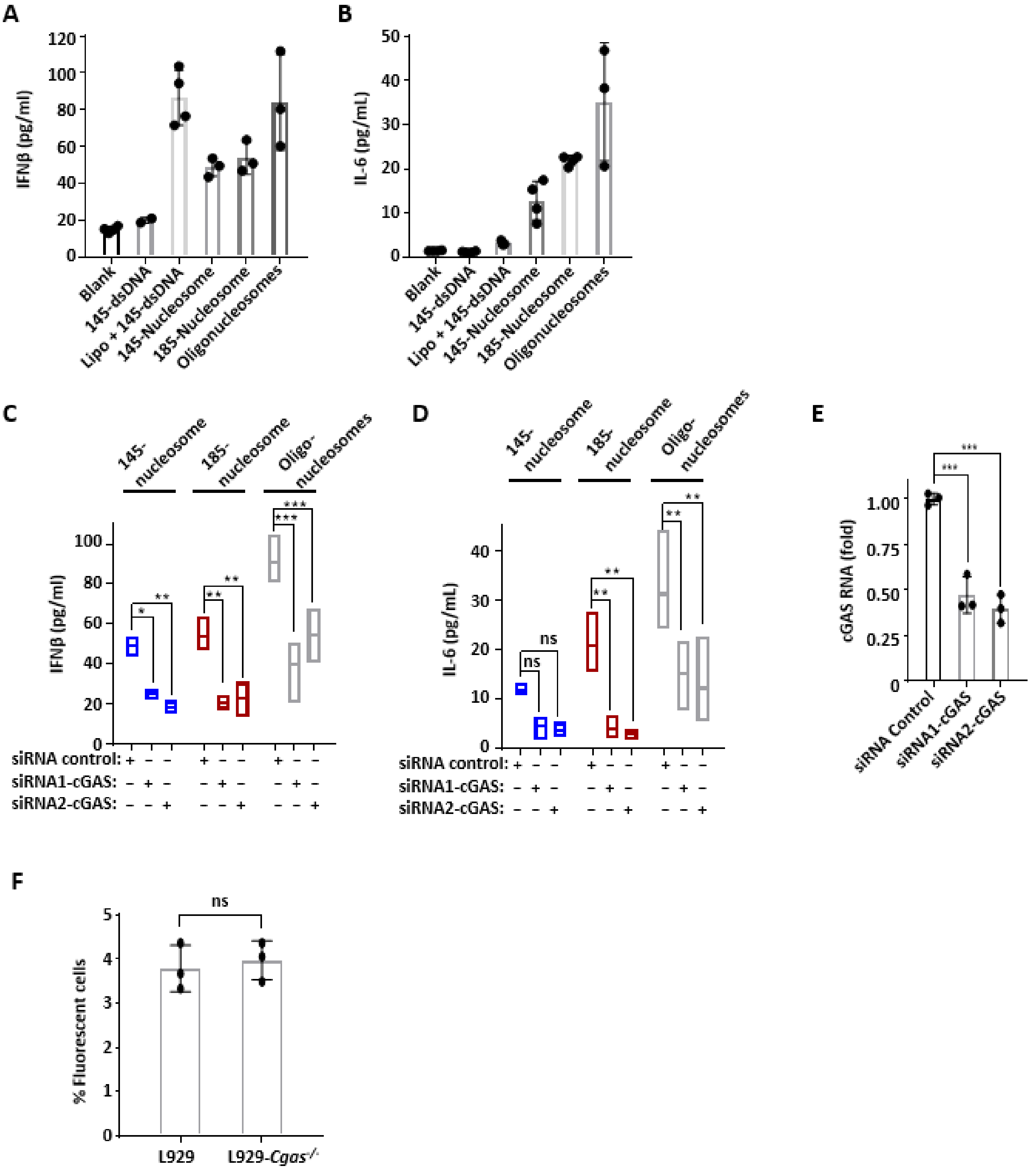

Supplementary Figure S12. Cellular uptake of nucleosomes stimulates the secretion of INFβ and IL-6 through cGAS activation. (**A-B**) ELISA analyses of IFNβ and IL-6 expression in THP1 cells upon incubation with different types of nucleosomes (20 nM) for 24 h. (**C-D**) ELISA analyses of nucleosome-induced IFNβ and IL-6 expression after cGAS knockdown by siRNAs. After treatment with siRNAs for 24 h, THP1 cells were incubated with 20 nM of nucleosomes for 24 h, and secreted IFNβ and IL-6 in the cell culture media were quantified based on ELISA analysis. (**E**) qRT-PCR analyses of cGAS

expression after cGAS knockdown by siRNAs. (**F**) Flow cytometry analyses of the uptake of 145-nucleosomes by L929 and L929-*Cgas*$^{-/-}$ cells. Cells were incubated with 145-nucleosomes (20 nM) for 6 h. For all graphs, error bars indicate mean ± standard deviation of three independent experiments. Statistical significance was determined based on Student's *t*-test (ns, p > 0.05; * 0.05 > p > 0.01; ** 0.01 > p > 0.001; *** 0.001 > p).

Supplementary Table S1. Primers used in the present study.

**Primers used for generation of 145- and 185-dsDNA molecules from the pGEM-3Z-601 vector template:**<br>Forward primer 145-dsDNA: 5'-(FAM)-CGATGTACGGGCCAGATATACG-3'<br>Reverse primer 145-dsDNA: 5'-TGGGCTATGAACTAATGACCCC-3'<br>Forward primer 185-dsDNA: 5'-(FAM)-CATATATGGGCTATGAACTAATGACC-3'<br>Reverse primer 185-dsDNA: 5'-TCAATAGGGGGCGTACTTGGCA-3'
**Primers used for the generation of 2450-dsDNA molecules from the pD-mNeonGreen-HA300-300 vector template:**<br>Forward primer 2450-dsDNA: 5'-(FAM)-CATCAGAGCAGCCGATTGTC-3'<br>Reverse primer 2450-dsDNA: 5'-CGCTACCAGCGGTGGTTTGT-3'
**Primers for RT-qPCR analysis:**<br>Forward primer H-IFNβ: 5'-AAACTCATGAGCAGTCT-3'<br>Reverse primer H-IFNβ: 5'-AGGAGATCTTCAGTTTCGGAGG-3'<br><br>Forward primer H-IL-6: 5'-GGTGTTGCCTGCTGCCTTCC-3'<br>Reverse primer H-IL-6: 5'-GTTCTGAAGAGGTGAGTGGCTGTC-3'<br><br>Forward primer H-ACTIN: 5'-TCATGAAGTGTGACGTGG-3'<br>Reverse primer H-ACTIN: 5'-CCTAGAAGCATTTGCGGT-3'<br><br>Forward primer H-cGAS : 5'-AAGGATAGCCGCCATGTTTCT-3'<br>Reverse primer H-cGAS : 5'-TGGCTTTCAGCAAAAGTTAGG-3'<br><br>Forward primer m-IFNβ : 5'-TCCGAGCAGAGATCTTCAGGAA-3'<br>Reverse primer m-IFNβ : 5'-TGCAACCACCACTCATTCTGAG-3'<br><br>Forward primer m-IL-6: 5'-CTGCAAGAGACTTCCATCCAG-3'<br>Reverse primer m-IL-6: 5'-AGTGGTATAGACAGGTCTGTTGG-3'<br><br>Forward primer m-ACTIN: 5'-ACCGGACAAGCTAAAGAAGGTGCT-3'<br>Reverse primer m-ACTIN: 5'-GCAGCAGGCGTTCCACAACTTTAT-3'
